# The Role of Phytochemicals and Gut Microbiome in Atherosclerosis in Preclinical Mouse Models

**DOI:** 10.3390/nu15051212

**Published:** 2023-02-28

**Authors:** Ann M. Centner, Leila Khalili, Vladimir Ukhanov, Saurabh Kadyan, Ravinder Nagpal, Gloria Salazar

**Affiliations:** 1 Department of Biomedical Sciences, Florida State University, Tallahassee, FL 32306, USA; 2 Department of Nutrition and Integrative Physiology, Florida State University, Tallahassee, FL 32306, USA

**Keywords:** *Akkermansia*, ApoE, atherosclerosis, berberine, CVD, gut, microbiome, polyphenols, TMAO

## Abstract

Gut microbiome alterations have recently been linked to many chronic conditions including cardiovascular disease (CVD). There is an interplay between diet and the resident gut microbiome, where the food eaten affects populations of certain microbes. This is important, as different microbes are associated with various pathologies, as they can produce compounds that are disease-promoting or disease-protecting. The Western diet negatively affects the host gut microbiome, ultimately resulting in heightened arterial inflammation and cell phenotype changes as well as plaque accumulation in the arteries. Nutritional interventions including whole foods rich in fiber and phytochemicals as well as isolated compounds including polyphenols and traditional medicinal plants show promise in positively influencing the host gut microbiome to alleviate atherosclerosis. This review investigates the efficacy of a vast array of foods and phytochemicals on host gut microbes and atherosclerotic burden in mice. Reduction in plaque by interventions was associated with increases in bacterial diversity, reduction in the *Firmicutes*/*Bacteroidetes* (F/B) ratio, and upregulation of *Akkermansia.* Upregulation in CYP7 isoform in the liver, ABC transporters, bile acid excretion, and the level of acetic acid, propionic acid, and butyric acid were also noted in several studies reducing plaque. These changes were also associated with attenuated inflammation and oxidative stress. In conclusion, an increase in the abundance of *Akkermansia* with diets rich in polyphenols, fiber, and grains is likely to reduce plaque burden in patients suffering from CVD.

## 1. Introduction

It is well known that the Western diet rich in simple sugars and saturated fat and low in fiber, vitamins, and minerals plays a large role in cardiometabolic syndrome (CMS), a global health issue [1,2]. CMS affects almost a quarter of the world’s population and involves both adverse cardiovascular and metabolic conditions such as hypertension, dyslipidemia, insulin resistance, systemic inflammation as well as central adiposity [2]. People suffering from CMS are at risk of developing type-2 diabetes (T2D) and cardiovascular disease (CVD) [3], the latter being a leading cause of death and disability [4]. A diet rich in plant foods offers protection for these conditions partially by influencing the host gut microbiota [5,6].

Investigation into human resident gut bacteria began with the realization that there are 10–100 trillion bacteria in our gut, which is 10 times greater than the number of human cells [7]. Emerging research has linked patients with many chronic conditions including CVD [8,9] and T2D [10] with an altered gut microbiome. In addition, various metabolites produced by resident gut bacteria are altered during disease. For example, trimethylamine N-oxide (TMAO) [11] and the short-chain fatty acid (SCFA) butyrate [6] are increased and decreased in CVD and T2D, respectively. Gut perturbations make the microbiome an attractive target for cardiometabolic health as it is linked to cholesterol transport, beneficial SFCA production, and vascular inflammation.

Microbiome dysbiosis is associated with reduced bacterial diversity and increased levels of disease-promoting bacteria, which is seen in many chronic inflammatory diseases. A feature of microbiome dysbiosis is the increase in the *Firmicute*/*Bacteroidetes* (F/B) ratio. These are the two most abundant phyla in the human gut. In animal models of CVD, nutritional interventions aimed at reducing atherosclerosis and its risk factors, like elevated total cholesterol (TC), low-density lipoprotein (LDL), and triglycerides (TG), reduce the F/B ratio, improve the lipid profile and lower inflammation. However, it is unclear whether the microbiome is the source of the protective effects seen in the vascular system. It is also incompletely understood the role specific genera/family of bacteria play in these effects. In this systematic review, we evaluated studies using foods/extracts rich in beneficial phytochemicals that reduced atherosclerotic plaque and microbiome dysbiosis in mouse models of atherosclerosis. In the following sections, we will provide an overview of the role of the microbiome in atherosclerosis and the beneficial effects of phytochemicals, including polyphenols, berberine, herbs used in traditional Chinese medicine, berries, grains, and fiber. The selected studies will then be discussed in depth with a focus on molecular mechanisms by which the microbiome and gut-derived metabolites regulate inflammation, lipid metabolism, and plaque accumulation in the aorta of mice. Through this analysis, we identified *Akkermansia* as one of the bacteria upregulated by many interventions that reduced plaque. These data suggest that dietary interventions that upregulate *Akkermansia* have the potential to improve the health of patients suffering from CVD.

## 2. Atherosclerotic Mouse Models

Apolipoprotein E (ApoE) is a fat-binding protein that helps regulate circulating lipid levels. It is important to note that while humans have three isoforms (E2, E3, and E4) of the ApoE gene, mice only have one ApoE isoform. ApoE (E3) is the most common isoform with almost 80% of ApoE found in this form. ApoE (E3) is considered a “wild-type” isoform, promoting normal plasma lipid levels in humans. On the other hand, ApoE (E2) and (E4) are associated with hyperlipidemias. Specifically, the E4 allele is linked to both CVD and Alzheimer’s disease [12]. Transgenic mice can be generated by homozygous replacement of ApoE with human ApoE (E3) and (E4) alleles, using mice on a C57BL/6J background [13]. Likely due to a myriad of reasons including greater expense, labor, and undesired side-effects of transgene alterations, these mice are not as widely used for research.

The ApoE knockout strain reviewed here was developed by Dr. Nobuyo Maeda [14]. Mice are homozygous for the ApoE^tm1Unc^ mutation and are on a C57BL/6J background. On the chow diet, ApoE^−/−^ mice have four to five times the circulating cholesterol of a wild-type mouse and develop intermediate atherosclerotic lesions after 15 weeks [15,16]. This process occurs more quickly on a high-fat or Western diet [15]. In contrast, wild-type mice require long-term high-fat or Western diet to develop atherosclerosis. Specifically, the ApoE^−/−^ mice develop severe cholesterol accumulation in macrophages, triggering a pro-inflammatory response and extracellular matrix (ECM) breakdown by cytokine and protease secretions, respectively [17]. Interestingly, the administration of a broad-spectrum antibiotic (ampicillin) in ApoE^−/−^ mice improved lipid profile and reduced atherosclerosis [18]. This demonstrates the role of the gut microbiome on atherosclerosis and in this particular mouse model.

The ApoE^−/−^ mouse model is the most common mouse strain used to study atherosclerosis and is also useful for studying other diseases such as Alzheimer’s disease [19] and respiratory diseases [20]. The second most common atherosclerotic mouse model is the LDL receptor (LDLR)^−/−^ mouse model. In contrast to ApoE^−/−^ mice, LDLR^−/−^ mice do not have hyperlipidemia on the chow diet and have higher basal HDL [21,22]. In addition, the LDLR^−/−^ model includes a less robust inflammatory response, fewer ECM changes, smaller aortic root lesions, and necrotic cores with fewer vascular smooth muscle cells (VSMCs) [21]. Overall, the atherosclerotic characteristics of the ApoE^−/−^ mice are more pronounced.

Reactive oxygen species (ROS) has been shown to accelerate atherosclerosis. A study overexpressing endogenous antioxidants in ApoE^−/−^ mice found that catalase and superoxide dismutase (SOD) resulted in smaller atherosclerotic lesions [23]. Previously, we reviewed many polyphenol-rich nutritional interventions (fruits, vegetables, nuts, grains, oils, spices, and teas) in atherosclerotic mice [24]. While several of the 73 studies reviewed found a reduced plaque burden and nine found no difference, the majority found a reduction in plaque in different portions of the aorta. While the proposed mechanisms of plaque reduction varied between interventions, they included improved lipid panel, improved antioxidant and inflammation status, and enhanced cholesterol clearance.

## 3. Microbiome and Atherosclerosis

**Figure 1:**
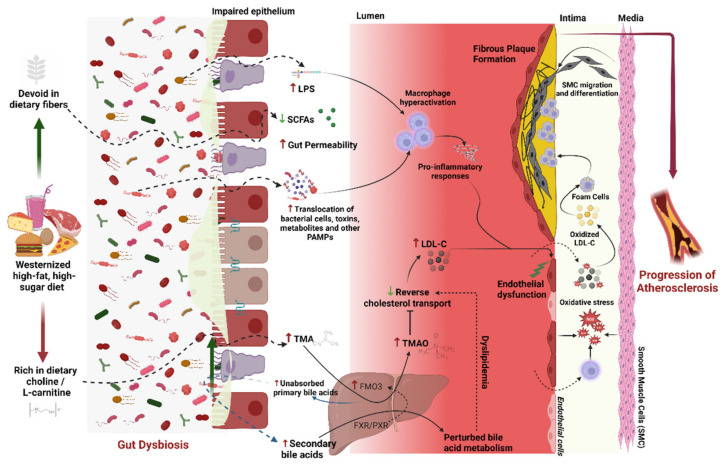
An overview of mechanisms and pathways via which the gut microbiome and its interaction with the host diet contribute to the pathophysiology of atherosclerosis. Intake of a westernized diet high in saturated fats and sugars instigates gut dysbiosis, which in turn triggers a cascade of various host metabolic pathways via the gut-liver-heart axis, eventually converging into atherosclerosis progression. Lack of fiber in these diets further lowers the production of microbiome-derived SCFAs leading to impaired gut epithelial permeability and translocation of microbes or their cell fragments (e.g., LPS), harmful metabolites, and PAMPs, which may provoke macrophages leading to excessive pro-inflammatory responses. On the other end, diets rich in choline/L-carnitine foster microbes harboring choline-TMA lyase enzyme leading to enhanced production of TMA and its conversion to pro-atherogenic TMAO via hepatic flavin-containing monooxygenase. TMAO impairs cholesterol metabolism by blocking reverse cholesterol transport leading to increased LDL-C. Besides, bile acid metabolism may also be perturbed by gut microbiome which is associated with dyslipidemia. Both these pathways may converge into endothelial dysfunction inducing oxidative stress by abnormal endothelial cells, activated macrophages and SMCs in the intima, and formation of foam cells via ROS-induced oxidation of LDL-C. This ultimately leads to arterial stiffness via bulging of fibrous plaque comprising of foam cells, differential SMCs, collagen, and elastin, thus marking the onset of atherosclerosis. Abbreviations: LPS: lipopolysaccharide; SCFAs: short-chain fatty acids; PAMs: pathogen-associated molecular patterns; LDL-C: low-density lipoprotein cholesterol; TMAO: trimethylamine N-oxide; FMO3: flavin-containing monooxygenase 3; FXR: farnesoid X receptor; PXR: pregnane X receptor; ROS: reactive oxygen species, SMC: smooth muscle cells. (↑): increased/higher, (↓): decreased/lower. Created with BioRender.com (accessed on 18 January 2023).

Recently, besides novel cellular and molecular mechanisms, there have been major advancements in the understanding of dietary, lifestyle, and environmental factors associated with the pathophysiology of atherosclerosis. One of the major elements linking these factors with cardiovascular health is the gut microbiome. Emerging evidence from clinical and animal experiments suggests that specific gut microbiome perturbations (‘gut dysbiosis’) may contribute to atherosclerosis and CVD predisposition and severity (Figure 1). Accordingly, remarkable progress continues to be made to elucidate mechanisms underlying or mediating the interplay between the microbiome, atherosclerosis, and cardiometabolic characteristics and to harness this knowledge to develop novel microbiome-based diagnostic and therapeutic avenues [25].

The gastrointestinal tract harbors a highly diverse and complex microbial community (‘gut microbiome’) comprising 10–100 trillion bacteria belonging to thousands of bacterial species that play an important role in our digestive, cardiometabolic, and immune health. These bacteria regulate the communication between the gut and other bodily niches by producing a plethora of metabolites some of which can translocate from gut into the blood circulation thereby regulating and influencing systemic health. Some of the prominent factors that modulate the diversity and composition of the gut microbiome community include diet, exercise, antibiotics, drugs, genetics, aging, and specific disease states. Abnormal alterations in the gut microbiome have been correlated with several diseases, including atherosclerosis [26,27]. In particular, specific microbial metabolites have been found to act as the mediators of atherosclerosis. Of these vasculotoxic and proatherogenic metabolites, the most classical and established example is TMAO, which is produced by hepatic oxidation of trimethylamine (TMA). This metabolite is derived from the gut bacterial metabolism of dietary choline and L-carnitine and promotes atherosclerosis by triggering platelet reactivity and vascular inflammation [28]. Studies have reported elevated levels of TMAO in people that consume diets rich in choline/carnitine (e.g., red meat) or have kidney disease [29]. In addition, some other microbiome-derived metabolites including phenylacetyl glutamine (derived from phenylalanine metabolism) and bile acids (derived from lipids) have also been found to play a role in atherosclerosis [27,30,31,32]. In contrast, certain microbiome-derived metabolites such as SCFAs (e.g., butyrate), which are produced primarily by the microbial fermentation of undigested dietary fibers in the large intestine, may prevent or ameliorate atherosclerosis by reducing inflammation and improving vascular functions [27,33]. This systematic review provides an update and discusses recent studies linking the gut microbiome and microbial metabolites with atherosclerosis, with special reference to dietary constituents rich in phytochemicals and polyphenols that may reduce atherosclerotic cardiovascular diseases by modulating the metabolic capacity and pathways of the gut microbiome.

## 4. Phytochemicals and Their Health Benefits

### 4.1. Polyphenols

**Figure 2:**
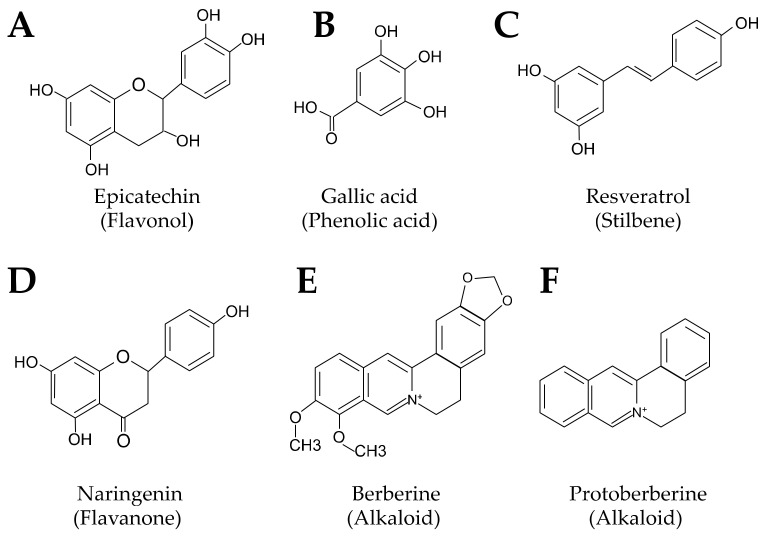
Structure of polyphenols and berberine. Examples of the structure of different types of polyphenols are shown for flavonols (**A**), phenolic acids (**B**), stilbenes (**C**), and flavonones (**D**). The alkaloid berberine (**E**) and the basic structure of protoberberines are also shown (**F**).

Plant-based foods such as fruits, vegetables, and whole grains are rich in fiber, vitamins, and minerals, as well as phytochemicals. Phytochemicals encompass compounds of plant origin including polyphenols and alkaloids. There are many types of polyphenols, and they are found in a variety of foods and beverages. Fruits, teas, wine, coffee, cocoa, and spices contain high amounts of polyphenols while vegetables, grains, legumes, and nuts contain lesser amounts [34]. Polyphenols can exert a number of functions including antioxidant, antimicrobial, and anti-inflammatory [34,35]. They can be further divided into flavonoids and non-flavonoids. Flavonoids include flavones, flavanones, flavonols or catechins, flavanols or flavab-3-ol, isoflavones, anthocyanidins, and chalcones [36]. They are characterized by the general chemical structure of a 15-carbon skeleton with two phenyl rings linked by a heterocyclic pyran ring (Figure 2A) [37]. Non-flavonoids include phenolic acids, stilbenes, and lignans. The chemical structure of non-flavonoids differs from flavonoids as it only contains one phenol ring, like gallic acid (Figure 2B). There are yet more specific substances in these categories, including resveratrol (a stilbene) (Figure 1C) and naringenin (a flavanone) (Figure 1D) [38]. Overall, phenolic acids, which are found in many fruits from berries to mangos as well as tea, coffee, and whole grains account for half of the daily intake of polyphenols [39].

Polyphenols became of interest to human health in the nineties; in 1993, they were correlated with reduced mortality from coronary heart disease (CHD) in an epidemiological study [40]. Subsequent in vivo and in vitro research solidified the correlative epidemiological findings. Polyphenols are potent antioxidants in vitro, but in humans, their effect is more complex as they undergo extensive modifications once ingested [41]. While the consumption of polyphenols in the human diet is approximately 1 g/day [39], it varies widely between individuals depending on the nutrient density of their diet. Despite the differences in intake, ultimately bioavailability is the key factor in the action of polyphenols.

Polyphenols are a heterogenous group, characterized by a structure as diverse as their metabolism. Digestion starts in the mouth with mastication and very minimal chemical digestion by the enzyme amylase. Next, polyphenols travel to the stomach where they are released with further chemical (pepsin) and mechanical (peristalsis) digestion. In general, bioavailability is low with approximately 5–10% of polyphenols being absorbed in the small intestine [42]. The simpler the structure, the greater the absorption at this step. Next, Phase I biotransformations including oxidation, reduction, and hydrolysis as well as phase II biotransformations including conjugation occurs in enterocytes [42]. The end results of these transformations are water-soluble metabolites containing methyl, glucuronide, and sulfate groups. From the small intestine, polyphenols can also travel in the circulation to the liver, where they undergo phase I and II metabolism. Next, polyphenols/their metabolites enter systemic circulation for tissue dissemination before traveling to the kidneys for urinary excretion [41,42]. The polyphenols not absorbed in the small intestine (90–95%) travel to the colon. Colonic bacteria break down the polyphenolic backbone and cleave glycosidic links [42]. Depending on the specific polyphenol, various end products are produced. Breakdown metabolites from flavonoids include lactones, aromatic acids and phenolic acids. From ellagitannins, which are non-flavonoid, free ellagic acid can be produced [42]. These metabolites can either be excreted through the urine or enter the portal circulation traveling to the liver for extensive phase II metabolism before delivery into the systemic circulation and to various organs. It is important to note that these metabolites can be more bioactive than their parent compounds. Examples include dihydroresveratrol (from resveratrol) and equol (from daidzein) [43]. Specific genera including *Clostridium* and *Eubacterium* are implicated in the metabolism of a vast number of phenolics [42]. Overall, the host bacteria influence what metabolites are produced.

Bioavailability of polyphenols depends on many factors including the food matrix and resident gut bacteria. Fiber, macro-, and micronutrients, as well as other phytochemicals in the meal, can influence polyphenol absorption either enhancing it or diminishing it [44]. Fiber and protein-rich foods as well as minerals (iron, zinc, calcium, magnesium) [45] can blunt polyphenol availability. On the other hand, carbohydrates, lipids, and other antioxidants can enhance polyphenol bioavailability. Further, as the gut microbiome is highly variable from person to person, so is their metabolism of polyphenols. This results in a high degree of interindividual variability to similar ingestion patterns of polyphenols [41].

Polyphenols and the gut microbiome interact in a bidirectional fashion. For example, the microbiota is required to break down polyphenols so they can become bioavailable, while phenolic compounds can alter the intestinal environment [43]. Phenolic compounds can improve gut health and balance by promoting the growth of beneficial bacterial families including *Bifidobacteriaceae* and *Lactobacillaceae* while restricting the growth of pathogenic bacteria such as *Escherichia coli* and *Helicobacter pylori*. Polyphenols can also increase certain beneficial species such as *Akkermansia, Prevotella*, and *Bacteroides* and reduce the ratio of two of the most predominant gut bacterial phyla F/B ratio [43]. Alterations in this ratio are a feature of microbiome dysbiosis and it is heightened in obese patients and those with metabolic syndrome [42] and CVD [46]. Overall, this review will touch upon many specific phytochemicals that change the gut microbiome and may be responsible for plaque reduction in atherosclerotic mice.

### 4.2. Berberine

While berberine is not a polyphenol, it is a bioactive alkaloid of plant origin (Figure 1E) known for its many beneficial effects including antioxidant, antimicrobial, anticancer, and antidiabetic [47]. Berberine belongs to the protoberberine group (Figure 1F), and it is composed of a quaternary ammonium salt of an isoquinoline alkaloid. Other members of the protoberberine group include berberrubine, jatrorrhizine, thalifendine, and columbamine. Berberine sources include tree turmeric, certain poppies, Chinese goldthread, barberry, and yellowroot [48]. Highly touted for its benefit for T2D patients due to its glycemic regulatory effects [49], it also shows promise for CVD patients due in part to its cholesterol-lowering effects [50].

Similar to polyphenols, berberine enters the oral cavity and is subject to chemical and mechanical digestion before traveling to the stomach and small intestine. From a study in rats, the bioavailability of berberine is reported to be very low at approximately 0.4% [51]. Due to the low presence of berberine in the plasma after ingestion, researchers hypothesized metabolites of berberine exert the beneficial effects associated with its ingestion. In line with this hypothesis is the fact that berberine is extensively broken down through phase I and phase II metabolism [51].

A recent review summarizes the many beneficial intestinal changes that occur with berberine supplementation [52]. In animals, these changes include a reduced diversity of microbiota as well as alterations in the relative abundance of *Desulfovibrio, Eubacterium,* and *Bacteroides*. In addition, reduced populations of *Mediterraneibacter gnavus, Blautia schinkii*, *Lactococcus lactis,* and several *Lactobacillus* strains were noted. Berberine has also been shown to increase SCFA (butyrate) production and reduce intestinal and systemic inflammation in animals. Lastly, berberine can activate the intestinal farnesoid X receptor (FXR), which plays a role in bile acid, lipid, and glucose metabolism and homeostasis.

### 4.3. Traditional Chinese Medicine

As one of the world’s oldest medicine practices, Chinese medicine has its roots 2000–3000 years ago and includes a robust use of medicinal plants for ailments. There are copious studies investigating the health benefits of traditional Chinese medicine. Common treatments arise during flu season and during the COVID-19 pandemic for their potential respiratory benefits. For example, *Echinacea* and Elderberry have been investigated for their protection against the common cold and for shortening the duration and/or intensity of symptoms. Although it should be noted that in a systematic review of the efficacy of *Echinacea* which included twenty-four double-blind clinical trials with over 4500 participants the evidence of these claims is weak [53]. A recent systematic review of five randomized trials using Elderberry concluded it may be a safe option for reducing both the duration and severity of colds [54]. *Ginkgo biloba* is another plant with origins in traditional Chinese medicine investigated for a number of ailments including asthma [55], Alzheimer’s disease [56], and metabolic syndrome [57]. More recently, the therapeutic attributes of Butterfly pea (*Clitoria ternatea*) fermented with symbiotic cultures of bacteria and yeasts revealed improvement in the markers of metabolic syndrome via modulation of gut microbiome in mice fed a cholesterol- and fat-enriched diet [58]. Overall, these examples demonstrate that it is important to investigate and critically evaluate whether traditional Chinese medicinal treatments are effective, rather than simply relying on their use throughout generations.

### 4.4. Berries

Whole fruits are a rich source of fiber and nutrients, including vitamins and minerals as well as polyphenols. Nearly 90% of Americans do not meet the daily fruit quota which is one and a half to two servings/day for women and men, respectively [59]. A recent review [59] summarizes the many beneficial health effects associated with fruit consumption. These include promoting gut health, promoting healthy weight, reducing risk for CVD and T2D, protection against certain cancers (colorectal and lung), promoting healthy aging, reducing severity of asthma and chronic obstructive pulmonary disease (COPD), and even improving psychological well-being.

Berries have many bioactive compounds which refer to phenolic compounds, flavonoids, and tannins. These compounds are in addition to nutritive carbohydrates, fiber, and vitamins and minerals. These bioactive compounds have been shown to have antioxidant, anti-inflammatory, and anticancer properties in human and animal studies [60]. They have also been shown to confer protection against CVD, Alzheimer’s disease, and depression [61], and alter the gut microbiome [62]. Specifically, 12-week *Aronia* berry whole fruit and extract supplementation in 66 healthy men was shown to improve vascular function and the extract increased *Anaerostipes* while the whole fruit increased *Bacteroides* [62].

### 4.5. Grains

Grains form the base of most diets and there are many different types ranging from rice to oats and wheat to ancient grains such as millet and quinoa. Today, the consumption of refined grains is high, despite the recommendation to intake half of the amount of grains each day as unrefined or whole [63]. Refining wheat refers to a milling process in which the bran and germ are removed. The bran is the portion of wheat that contains the highest levels of polyphenols and fiber which have both been shown to exert an array of health benefits [64,65]. A recent review highlights that higher consumption of whole grains is linked to a lower risk of CVD, diabetes, obesity, and certain gastrointestinal conditions [65].

### 4.6. Fiber

Dietary fiber has been linked to many positive healthy outcomes through epidemiological studies [66]. Dietary fiber is abundant in whole plant foods such as fruits, vegetables, legumes, and whole grains. However, the current American diet includes many sources of refined grains and fruit juices instead of their fiber-containing counterparts [59,67]. Increasing dietary fiber is associated with improved metabolic health, namely through targeting insulin sensitivity pathways, and promoting a healthy body weight [67]. Other benefits of fiber include reduced CVD and T2D risk, improved colonic health and gut motility, and reduced incidence of colorectal cancer [67]. Higher intake of fiber is even correlated with increased lifespan. Recommended daily intake of fiber is 22–28 g/day and 28–34 g/day for women and men, respectively, according to the 2020–2025 Dietary Guidelines for Americans [63]. However, according to National Health and Nutrition Examination Survey (NHANES) data, the average intake is 50% below the recommended intake [68]. Interestingly, many Americans realize fiber is important to overall health, yet believe they consume enough [69].

### 4.7. Sterols

Plant sterols, also known as phytosterols, have been implicated in promoting overall health by reducing the risk of chronic cardiometabolic diseases [70]. These are cholesterol homologs that are an integral component of many unrefined plant oils such as olives, sesame, almonds, safflower, soyabean, and peas [71]. Sitosterol and campesterol are the major phytosterols in foods, comprising about 60% and 35%, respectively. Owing to their low intestinal absorption (0.5–4%), their concentration in the human body is 1000 times less than cholesterol [72]. Besides, these functional molecules could competitively inhibit intestinal absorption of cholesterol [73]. Moreover, maternal supplementation of functional sterols could also improve the immunity in breastfed infants via positively modulation of lipid profiles and gut microbiome composition of breast milk [74].

## 5. Phytochemicals in Atherosclerosis and the Gut Microbiome

### 5.1. Data Extraction

The literature search was conducted using Web of Science, Science Direct, PubMed, and Embase until November 2022. The following search terms were used: (ApoE^−/−^ OR LDLR^−/−^) and (Mice OR Mouse) and (Gut microbiota OR gut microbiome) AND (Atherosclerosis) AND (Plaque OR lesion) AND (Nutrition). Our search was restricted to studies published in the English language.

The following criteria were used to identify eligible studies: (i) animal studies performed on mice and (ii) investigation of the effects of nutritional interventions on gut microbial composition and atherosclerotic plaque. Exclusion criteria were (i) lack of sufficient information on end-trial findings and (ii) review studies, meta-analyses, and any other type of article that is not an original research study.

**Figure 3:**
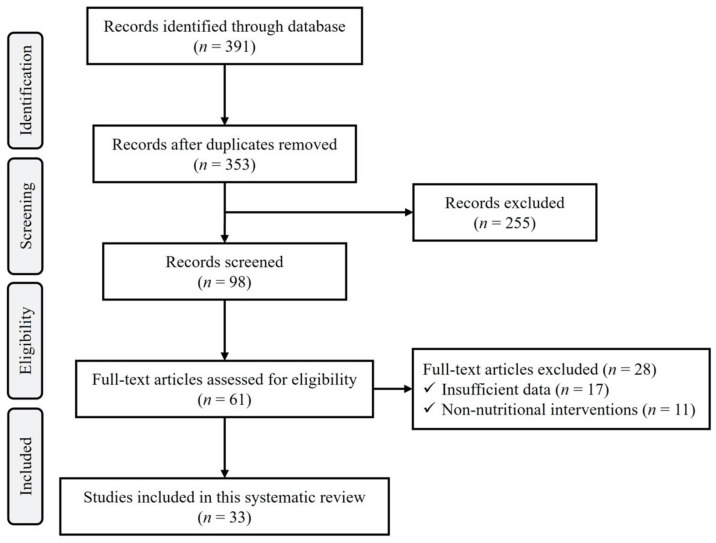
PRISMA flow diagram. Studies were identified through databases search in PubMed, Embase, Web of Science, and Science Direct. Of the 420 studies identified, only 62 were included in this systematic review.

For this systematic review, the search process identified 420 articles for potential inclusion. In total, 127 manuscripts contained original research data. After reviewing the titles and abstracts, 90 studies met the initial inclusion criteria. Following the initial selection, 28 studies were excluded, mainly because they did not contain complete data, or the study did not investigate the direct effect of nutritional intervention on gut microbiota and atherosclerosis (Figure 3).

Overall, 33 papers met the inclusion criteria. Among the included studies 15 evaluated the effect of plant extracts, four evaluated the effect of plants (such as tea-like plants, dietary fruit and vegetable, brown bean, and Brussels chicory), four evaluated the effect of plant juice (such as raw garlic juice, *Alisma orientalis* beverage, and pomegranate juice), five evaluated the effect of traditional Chinese plant mixtures (such as Xin-Jie-Yu granule, polygoni multiflori radix, dingxin recipe, red yeast rice, and TongMai ZhuYu), two evaluated the effect of spices (such as curcumin), one evaluated the effect of fiber supplementation, eight focused on the effect of berries, two studied the effect of poly- and oligo-saccharide, 12 evaluated the effect of microbes (probiotics), and one evaluated the effect of gut microbiota-driven peptides on gut microbiota composition and atherosclerosis plaque in atherosclerotic mice models.

### 5.2. Characteristics of Included Studies

The two common mouse models used to study atherosclerosis or the plaque build-up occurring during CVD are the LDLR^−/−^ and ApoE^−/−^. The latter is utilized more extensively, thus the majority of the papers in this review use ApoE^−/−^ mice, which are on a C57BL/6 (wild-type mouse) genetic background. Of the 33 studies, the majority used ApoE^−/−^ mice, whereas only a few studies used LDLR^−/−^ mice. Additionally, most of the studies used males (31 studies), and only 2 used both sexes. Our review will highlight our study [75] which used both males and females as well as one other study using both sexes [76]. Many studies had either a control group comprised of wild-type (C57BL/6) mice and/or a control group fed a regular chow diet, and an additional model group fed a high-fat diet (HFD). Changes will be described in relation to atherosclerotic mice on HFD if treatment is in tandem with HFD. If all mice are on the chow diet, then changes will be made to mice without additional nutritional intervention.

The majority of the studies observed positive correlations in which improvement in microbiome dysbiosis was associated with reduced atherosclerosis. Only a few studies directly evaluated the role of the microbiome using either antibiotics or fecal transplantation.

Of the 33 studies, 19 were identified in categories of most interest which included polyphenol extracts and foods rich in polyphenols such as berries and grains. Included in these 19 were the phytochemical berberine and plant-derived traditional Chinese medicines. Six of the included papers either did not assess either atherosclerosis [77,78] or the gut microbiome [79,80] or found no reduction [81,82] with the intervention. Of note, our gallic acid paper only observed a reduction in males, but not females [75]. The other study using males and females did not report sex differences. Fifteen of these studies utilized male mice exclusively [77,78,81,83,84,85,86,87,88,89,90,91,92,93,94] while eight studies used only female mice [95,96,97,98,99,100,101,102]. Three studies [82,103,104] did not report the sex of mice used.

### 5.3. Polyphenols

There are seven polyphenol studies [75,79,89,96,98,102,103] that examined plaque burden in correlation with gut microbiome changes. These studies included a mix of sex and animal model types. One study [103] used 12–13-week-old LDLR^−/−^ mice (*n* = 12/group), and sex was not disclosed. The rest of the studies used ApoE^−/−^ mice. Our study [75] used both males and females (three to four months old, *n* = eight/group/sex). Three studies used females (*n* ≥ eight/group) exclusively. Two of these female mice studies used seven to eight-week-old mice [96,102], while one [98] did not disclose the age of the mice. Two studies used males exclusively, with one using three-week-old mice (*n* = 15/group) [89] and one using seven-week-old mice (*n* = six/group) [105].

Nie et al. [103] examined the effects of quercetin in LDLR^−/−^ mice. Mice were fed a regular chow diet for four weeks then switched to HFD (45% fat) for eight weeks. A quercetin solution (100 µg/day) containing 1% sodium lauryl sulfate was provided to half the mice. Quercetin attenuated the HFD-induced weight gain in the mice and plaque burden, which was measured in the aortic sinus. Quercetin appeared to combat oxidative stress and inflammation by reducing malondialdehyde (MDA) and interleukin (IL)-6 levels. Plasma inflammatory markers including tumor necrosis factor alpha (TNF-α), interleukins (IL-10 and IL-17), and monocyte chemoattractant protein-1 (MCP-1) were also assessed but unchanged by treatment. Intestinal atherogenic lipid metabolites were assessed and lysophosphatidylcholines (LPA18:1, LPA18:2, LPA20:4), and oxidized phospholipids (PEIPC, POVPC, and PGPC) were reduced with quercetin. In addition, quercetin reduced total cecal bile acids and cholesterol and increased coprostanol, a cholesterol derivative. Quercetin increased alpha-diversity and decreased the F/B ratio while increasing the relative abundance of *Actinobacteria*. Genus level changes included increased abundances of *Akkermansia, Bacteroides, Parabacteroides,* and *Ruminococcus* and decreased *Lactobacillus*.

Two polyphenol studies, using resveratrol [96] and geraniin [98], found correlations between plaque attenuation and changes in TMAO pathways. Chen et al. fed mice a normal chow diet with or without resveratrol (0.4%) in the presence or absence of antibiotics for 16 weeks. In contrast, Lin et al. fed mice a low or high choline diet (0.08% and 1%, respectively). Geraniin was dissolved in drinking water and provided at a dose of 80 mg/kg body weight (BW)/day for 12 weeks along with 1% choline.

Resveratrol and geraniin increased flavin-containing monooxygenase 3 (FMO3) protein levels in the liver. In addition, both treatments decreased blood levels of TMAO. The mechanism proposed for a reduction in TMAO by resveratrol was a decrease in gut bacteria that produce TMA. Importantly, the study supplementing mice with resveratrol reported that with antibiotics and treatment, TMAO was not attenuated. To study the mechanism of action of geraniin, in vitro work in macrophages (RAW264.7) suggests changes in the scavenger receptor cluster of differentiation 36 (CD36), transmembrane protein 106a (TEM106A), apolipoprotein C1 (APOC1), macrophage scavenger receptor types (MSR) I and II, and alpha-2-macroglobulin are responsible. Both studies observed a decrease in the F/B ratio. Resveratrol elevated the relative abundances of *Bacteroides, Lactobacillus, Bifidobacterium,* and *Akkermansia* and decreased the relative abundances of *Prevotella, Ruminococcaceae_unclassified,* and *Biophila*. Geraniin increased the relative abundance of bacterial genera *Bacteroides, Alloprevotella,* and *Alistipes*. Thus, both polyphenols increased *Bacteroides*. Interestingly, geraniin in combination with choline and antibiotics increased *Akkermansia*. Inflammatory plasma molecules were also measured with the addition of geraniin to the diet. An increase was observed for IL-10 (anti-inflammatory cytokine), while IL-1β, IL-6, and TNF-α were reduced. In the resveratrol study, enhanced bile acid deconjugation and fecal excretion were observed. Specifically, with treatment, there was a decrease in fecal conjugated/unconjugated bile acid ratio and an increase in bile salt hydrolase enzymatic activity. This study examined the enterohepatic FXR-fibroblast growth factor 15 (FGF15) axis by examining the ileal protein and mRNA levels of key players. This axis maintains bile acid homeostasis and findings indicated the enterohepatic FXR-FGF15 axis played a key role in resveratrol-induced bile acid synthesis. Importantly, these changes with treatment were not observed when mice received antibiotics and treatment. Bile acid-related mechanisms were not assessed in the geraniin study.

Two studies by Ghosh et al. and Zhang et al. [79,105] assessed the efficacy of curcumin in atherosclerosis and gut health in mice. The study by Ghosh et al. was excluded as it did not assess gut bacteria changes. Zhang et al. [105] treated seven-week-old ApoE^−/−^ mice (*n* = 6/group) with cadmium for three months to investigate plaque burden and gut microbiome changes. Cadmium was dissolved in drinking water and applied at low and high doses (100 and 200 mg/L) with both the chow diet and HFD groups. Aortic roots were stained with Oil Red O to assess lipid burden. With cadmium and HFD, the aortic plaque was increased in correlation with gut microbiome changes including reduced bacterial diversity and changes in composition as well as enhanced TMAO synthesis. The pathology of cadmium was confirmed with fecal transplantation from a cadmium-exposed mouse to a non-cadmium-exposed mouse. In addition, curcumin was delivered to mice at increasing concentrations (100 and 200 mg/kg/BW) via gavage daily for two months after one month of cadmium exposure. The curcumin group was fed HFD and compared to the HFD group also exposed to cadmium. Curcumin was shown to reduce plaque burden and remodel the gut microbiome. Curcumin also reduced plasma lipids including TC, TG, and LDL, while increasing HDL. Interestingly, curcumin reduced cadmium blood and urinary concentrations. In addition, curcumin affected inflammation status, notably by reducing aortic protein expression of nuclear factor kappa B (NF-κB) p65 and NLR family pyrin domain containing 3 (NLRP3) and concentrations of IL-1β and IL-6. Compared to HFD alone, curcumin reduced plasma TMAO, the F/B ratio, the *Lactobacillaceae* family, *Unspecified_S24_7,* and *Lactobacillus* abundances, and increased the abundance of *Verrucomicrobia* and *Akkermansia*. A shift in aortic root macrophage polarization exhibited by increased M2-type polarization and decreased M1-type polarization with curcumin was also observed by immunofluorescence and flow cytometry. M1-type polarization in macrophages can be promoted by TMAO through NLRP3 activation [106] and has been shown to decrease aortic plaque stability [107]. Overall, Zhang et al. [105] demonstrated that cadmium exposure worsens atherosclerosis through gut microbiome perturbations, heightened TMAO synthesis, and M1-type macrophage polarization. Further, curcumin was found to reduce these cadmium-induced changes.

Yang et al. [89] treated 3-week-old male ApoE^−/−^ mice with procyanidin A2, a polyphenol belonging to the proanthocyanidin class of flavonoids, which are the second most abundant type of natural phenolic [108]. Mice were treated with and without procyanidin A2 (110 mg/kg BW), which was added to drinking water and applied along with HFD (21% fat, 0.15% cholesterol) with and without antibiotic mix (1 g/L neomycin sulfate, 1 g/L metronidazole, 1 g/L ampicillin, and 0.5 g/L vancomycin) in drinking water. Treatment was administered for 12 weeks, and results were compared to mice receiving HFD alone. Procyanidin A2 reduced HFD-induced plaque accumulation, although this paper failed to include plaque area quantification. Interestingly, Yang et al. concluded that antibiotics plus procyanidin A2 almost offset the attenuation of atherosclerosis seen with procyanidin A2 alone. Again, quantitative data is missing, but aortic sinus lesion areas stained with Oil Red O and hematoxylin and eosin (H&E) counterstain are provided. On the other hand, macrophage accumulation in the aorta was both depicted with cross-section staining of CD68 and quantified. Procyanidin A2 without antibiotic lowered macrophages. In addition, procyanidin A2 decreased HFD-induced TC, LDL, and increased HDL. TG was unaffected by polyphenol, but the addition of antibiotic (plus procyanidin A2) resulted in a decrease in TG. In terms of oxidative stress burden, there was no change in SOD with procyanidin A2 addition, but MDA was reduced without antibiotic. In addition, procyanidin A2 alone (without antibiotics) reduced adhesion molecules (ICAM-1 and VCAM-1) in aortic tissue. These molecules mediate the migration and adhesion of inflammatory cells to promote atherosclerosis. For mRNA expression of hepatic lipid metabolism genes, procyanidin A2 alone increased peroxisome proliferator-activated receptor γ (PPARγ), cholesterol 7 alpha-hydroxylase (CYP7A1), and ATP-binding cassette subfamily A member 1 (ABCA1). Lastly, the gut microbiome was assessed with 16S rRNA sequencing. Procyanidin A2 led to a substantial increase in biodiversity while the addition of antibiotics led to a great reduction in biodiversity. Procyanidin A2 also decreased the F/B ratio, while increasing the relative abundances of *Verrucomicrobia, Akkermansia, unclassified_f__Prevotellaceae*, and *Coriobacteriaceae_UCG-002*. Targeted MS metabolomics analysis of plasma from procyanidin A2-treated mice identified four procyanidin A2-derived phenolic acid metabolites, including hydroxyphenylacetic acid (HPAA), hydroxyphenylpropionic acid (HPPA), 3,4-dihydroxyphenylpropionic acid, and 3,4-dihydroxyphenylacetic acid. HPPA, the major metabolite, has been shown to prevent lipid accumulation and inflammation, thus preventing foam cell creation from macrophages. The addition of antibiotics led to massive decreases in HPAA and HPPA suggesting the gut microbiome is crucial to the breakdown of procyanidin A2. In conclusion, the microbiome and polyphenol procyanidin A2 have a symbiotic relationship. Specifically, the microbiome is crucial to metabolize procyanidin A2 for this polyphenol to exert beneficial effects on aortic plaque, oxidative stress, inflammation, and lipid metabolism.

Naringin is a flavonoid found in citrus fruit with potential to interact with gut bacteria and alleviate atherosclerosis. A study using seven-week-old female ApoE^−/−^ mice (*n* = eight/group) investigated the efficacy of daily naringin (100 mg/kg BW) supplementation with HFD [102]. There was also a group treated with 10 mg/kg/day of atorvastatin, a cholesterol-lowering drug, in 0.5% sodium carboxymethyl cellulose. Aortic plaque was assessed in the whole aorta as well as in aortic sinus cross sections, both stained with Oil Red O. Similar to the statin, plaque in the whole aortic and aortic sinus was significantly reduced with naringin. Naringin lowered serum lipids similar to the statin with specific decreases occurring for TC and LDL. Naringin and the statin also reduced serum total bile acids and liver TC, while naringin alone reduced liver TG. Serum HDL and oxidized (ox)-LDL were not affected by the statin or naringin. Nontarget metabolomics were used to assess lipid metabolites in the liver. Naringin reduced liver amounts of TG, sphingomyelin, diglyceride, phosphatidic acid, phosphatidylcholine, and glycerophospholipid. The greatest polyphenol-induced decreases occurred for cholesterol ester and campesterol 6′-(9Z-octadecenoyl)-glucoside. The decrease in cholesterol ester may explain the decrease in serum TC, as cholesterol ester is the main component of serum cholesterol. Naringin also reduced the levels of cholic acid, tauroursodeoxycholic acid (TUDCA), ursodeoxycholic acid (UDCA) and glycocholic acid. Glycine and taurine, which assist in the conjugation of bile acids trended toward a decrease with naringin suggesting enhanced excretion of conjugated bile acids. Kyoto Encyclopedia of Genes and Genomes (KEGG) analysis showed that naringin affected pathways associated with energy and carbohydrate metabolism and bile acid and steroid synthesis. Overall, naringin may increase bile acid excretion through enterohepatic circulation resulting in a decrease in serum lipids. In line with this, naringin increased total lipid excretion in feces and altered gut microbiota associated with cholesterol metabolism. Surprisingly, naringin and the statin increased the F/B ratio and decreased *Verrucomicrobia*. Naringin also reduced the relative abundances of *Bacteroides, Bifidobacterium, Lactococcus*, and *Clostridium sensu_- stricto_1.* These changes indicate this polyphenol suppresses hydrolysis of conjugated bile acids and dampens the FXR/FXF15 axis resulting in intestinal bile acid accumulation. In fact, mRNA levels of FXR and FGF15 in the ileum were significantly reduced in mice exposed to naringin. On the other hand, increases in the abundances of 7α-dehydroxylase-producing bacteria, *Eubacterium_fissicatena, Eubacterium_coprostanoligenes*, and *Eubacterium_brachy* were observed with naringin suggesting enhanced degradation of free bile acids. *Eubacterium* converts cholesterol into coprostanol, which was found to be increased in the mice feces exposed to naringin. Stimulation of the FXR/FXF15 axis has been shown to dampen hepatic cytochrome P450 enzyme expression. In line with this, mRNA levels of cytochrome P450 enzymes CYP7A1 and CYP7B1, which are key genes involved in bile acid biosynthesis, were significantly increased by naringin. Relative levels of hepatic genes involved in cholesterol metabolism were assessed. Naringin decreased proprotein convertase subtilisin/kexin type 9 (PCSK9) and inducible degrader of low-density lipoprotein receptor (IDOL), which are both involved in TC reverse transport. Lastly, similar to the statin, naringin reduced plasma TMA and TMAO. Overall, the mechanism of reducing plaque burden by naringin involves gut microbiome remodeling and subsequent cholesterol metabolism changes.

Recently, our lab investigated the effects of gallic acid (0.2% in drinking water) in male and female ApoE^−/−^ mice (*n* = eight/group/sex) [75]. The treatment duration was seven weeks and mice were fed a chow diet for two weeks, then HFD for five weeks. This polyphenol was chosen based on our earlier finding that blackberry plus HFD reduced plaque burden in male, but not in female mice, independent of blood lipid changes. Likewise, only male mice responded to gallic acid treatment with significantly reduced plaque assessed in the whole aorta by *en face* analysis. This plaque reduction occurred in both the arch and descending portions of the aorta. Circulating lipids did not correlate with plaque reduction, but inflammation status was correlated. Specifically, males had a reduced spleen weight and serum IL-3 and IL-12 levels and an improvement in gut health. Gut changes induced by gallic acid included downregulation of HFD-induced increases of *Eubacterium fissicatena*, *Turicibacter* and *Dorea* as well as upregulation of *Akkermansia* assessed by 16S rRNA metagenomics in male feces. Gallic acid did decrease the HFD-induced upregulation of the F/B ratio in both sexes, but this finding did not reach significance. In contrast, in females, *Akkermansia* was downregulated together with *Dorea*. Still, it can be concluded that gallic acid partially restored gut dysbiosis. Overall, these finding suggests gallic acid may alleviate atherosclerosis in a sex-dependent manner by modulating the gut microbiome. Since the lack of effect in females correlated with reduced *Akkermasia* instead of upregulation as seen in males, it is possible that upregulation of *Akkermasia* is required for gallic acid-plaque reducing effects.

### 5.4. Berberine Reduces Plaque and Remodels the Gut Microbiome

Four studies observed plaque reduction with berberine (12–16 weeks) and all of these studies used 6–8-week-old ApoE^−/−^ mice (*n* = 8–12 per group), while two used males [87,88] and two used females [99,100]. All studies had a control group (which sometimes were wild-type/C57BL/6J mice) on normal chow diet which is typical for many of the studies included in this paper. Since these groups failed to develop substantial aortic lesions, comparisons (i.e., treatment effect) were made to the atherosclerotic mouse model and HFD groups. Three studies used HFD [87,88,100] while one study used a high-choline diet [99]. Choline is found naturally in red meat, eggs, and dairy. Choline is converted to TMA by gut bacteria then TMAO by the enzyme FMO3 in the liver. TMAO is associated with CVD risk and promotes atherosclerosis [109]. Importantly, all of these berberine studies observed plaque attenuation correlated with gut microbiome changes, but the dosing strategies, method of plaque analysis, and specific bacteria alterations differed.

For dosing, two groups had low and high treatments of berberine. Li et al. [99] added berberine to the diet at 100 mg/kg and 200 mg/kg while Wu et al. [87] administered berberine by gavage (once daily) at 50 mg/kg and 100 mg/kg. Shi et al. [88] also administered berberine by gavage (50 mg/kg, only twice weekly) and lastly, Zhu et al. [100] added berberine in drinking water (0.5 g/L). Li et al. and Shi et al. reported similar body weights between groups, while the two other studies did not report on this measure.

For plaque analysis in the aorta, three studies [99,100,109] examined the whole aorta *en face* stained with Oil Red O for lipid deposition. These three studies and the last berberine study [88] examined sections of the aorta for plaque as well (H&E and Oil Red O staining). The last study had a berberine treatment group co-housed with non-treated animals and found reduced lesion area in both groups. In addition, only this study [88] analyzed collagen in the plaque (Masson’s trichrome staining), finding a significant decrease with berberine treatment in the co-housed group.

Wu et al. [87] reported the F/B ratio as unchanged, while Shi et al. [88] observed an increase in the F/B ratio. Li et al. [99] and Zhu et al. [100] did not report on the F/B ratio. Two studies observed an increase in the phylum *Verrucomicrobia* [88,100], with one of these studies noting an increase in the main genus of this phylum, *Akkermansia.* Zhu et al. [100] also observed an increase in *Bacteroides* (genus), while Shi et al. [88] observed a decrease in phylum *Proteobacteria*. Li et al. [99] observed an increase in the abundance of *Lachnospiraceae NK4A136 group, Bacteroidales S24-7 group* (unclassified), and *Eubacterium, Marvinbryantia, Clostridiales unclassified, Ruminiclostridium 5, Prevotellaceae NK3B31, and Bifidobacterium.* Wu et al. [87] observed an enrichment of the abundance of *Roseburia, Blautia, Allobaculum, Alistipes, and Turicibacter*.

### 5.5. Traditional Medicine

#### 5.5.1. Gingko Biloba

In terms of the scope of this paper on atherosclerosis and gut health, *Ginkgo biloba* was investigated in two studies using six- to eight-week-old male atherosclerotic mice. One study used ApoE^−/−^ mice and a six-week intervention [91] while the other used LDLR^−/−^ mice and a 12-week intervention [94]. Wang et al. [94] used *Ginkgo biloba* leaf extract comparing it to a defined herbal preparation (EGb761). Lv et al. [91] used Ginkgolide B, extracted from *Ginkgo biloba*, and compared it to Atorvastatin. Studies used either Western diet (41 kcal% fat and 0.21% cholesterol) [94] or HFD [91] for the intervention groups and administered the treatments via gavage. Interestingly, both studies improved the lipid profile and inflammation status of the mice in correlation with reduced plaque burden and gut microbiota changes.

It is important to note that plaque burden was assessed using different methods, as Lv et al. [91] examined both the whole aorta and aortic root cross sections while Wang et al. [94] only assessed cross sections. Both studies utilized Oil Red O and H&E staining. Lv et al. [91] also used Masson staining but did not report on findings related to this as the cross-sections appear to have been visual aids and whole aortas utilized for plaque reduction quantification purposes. Both studies found that the treatments reduced plaque similar to the control treatment (i.e., EGb761 and Atorvastatin).

Remarkably, Lv et al. [91] found that Ginkgolide B reversed all HFD-induced lipid accumulations, besides HDL which is considered beneficial as it shuttles cholesterol to the liver from the bloodstream. Specifically, serum reductions in TC, TG, LDL, very-low-density lipoprotein (VLDL), and high-sensitivity C-reactive protein (hs-CRP) and increased HDL were noted. Decreases in liver TC and TG and blood glucose were also observed. A limitation of this study is that pro-inflammatory cytokines and interleukins were not examined, thus CRP is the only measure of acute inflammation in this study. Similarly, Wang et al. [94] found that *Gingko biloba* decreased the Western diet-induced lipid accumulation namely by reducing TC, LDL, and TG levels. In addition, arterial transcriptional levels of the macrophage marker CD68 and scavenger receptors (CD36 and scavenger receptor a1, SR-A1) were reduced with *Gingko biloba*, indicating inhibition of macrophage cholesterol uptake. *Gingko biloba* also reduced serum levels of pro-inflammatory molecules including MCP-1, IL-1β and TNF-α.

Lv et al. [91] examined TMA/TMAO and FMO3 finding that Ginkgolide B inhibited FMO3 mRNA and protein expression in the liver and lowered TMA and TMAO levels in both plasma and kidney samples. While Wang et al. [94] did not report on TMAO, this study did measure bile acids, Indole-3-acetic acid (IAA), SCFAs, and intestinal barrier function-related proteins. Findings indicated *Ginkgo biloba* improved gut health by increasing serum secondary bile acids while reducing primary bile acids and IAA. SCFAs and tight junction proteins (claudin-1 and zonula occludens-1, ZO-1) were upregulated. Specifically, serum concentrations of many SCFAs including formic acid, acetic acid, butyric acid, valeric acid, and hexanoic acid were increased with *Ginkgo biloba*. In vitro in lipopolysaccharides (LPS)-exposed human monocytes (THP-1 cells), IAA was associated with a decrease in pro-inflammatory mRNA expression of IL-1β, IL-6, and TNF-α.

In the gut, Lv et al. [91] found that Ginkgolide B decreased the F/B ratio and decreased *Deferrobacters*. At the genus level, Ginkgolide B increased *Bacteroides* and decreased *Helicobacter* and *Roseburia*. Changes in *Bacteroides* and *Helicobacter* were seen in 16S rRNA and RT-qPCR and were associated with improvements with Ginkgolide B supplementation. Wang et al. also observed a decreased F/B ratio with *Gingko biloba*. In addition, *Gingko biloba* supplementation increased the levels of *Desulfovibrionaceae, Akkermansia, Alistipes, Rikenellaceae RC9 group, Alloprevotella and Parabacteroides. Gingko Biloba* reduced the abundances of taxa under the *Firmicutes* phylum including *Blautia, Lachnospiraceae, Lachnoclostridium, Colidextribacter, Faecalibaculum, Roseburia, Dubosiella, Erysipelatoclostridium* and *Ruminococcus torques* group. Overall, the two changes found in the microbiota with Ginkgolide B and *Gingko Biloba* supplementation included a decrease in F/B ratio and *Roseburia* abundance. In addition, as seen in several berry studies, *Gingko Biloba* increased *Akkermansia*.

Lastly, Wang et al. [94] performed a fecal microbiome transplant to demonstrate whether the microbiome of *Ginkgo biloba* supplemented mice could reduce plaque burden. This study did find that a microbiome transplant via gavage could reduce plaque burden.

#### 5.5.2. Tea

Two studies from China investigated the effects of tea plants/their polyphenols. One study examined the effect of *Ligustrum robustum*, the plant used to make Ku-ding tea. The second study investigated the effects of tea polyphenols. Both studies used eight-week-old ApoE^−/−^ mice (*n* = 10/group) with a study duration of 16–17 weeks, but the other study’s design parameters were different. Liu et al. [101] used only female mice, while Liao et al. [76] used both males and females. In addition, the *Ligustrum robustum* was delivered by gavage in mice on a high choline diet. The 700 mg/kg/day dosage delivered was reported to be equivalent to 5 g tea per day for a 60 kg adult. The mice in the tea polyphenol study by Liao et al. were fed HFD, and increasing amounts of polyphenols (0.4, 0.8, 1.6 g/L) were added to the drinking water along with sucrose (30 g/L) to mask the bitterness. Liao et al. reported no changes in body weight between HFD and HFD plus tea polyphenols.

In line with previous papers, Liu et al. [101] examined TMA, TMAO, and FMO3. *Ligustrum robustum* reduced serum TMA and TMAO but had no effect on liver gene and protein expression of FMO3. Liao et al. [76] did not measure these markers but did measure circulating lipids, finding that tea polyphenols reduced TC, LDL, and HDL. The reduction in HDL was partially explained by a decrease in total cholesterol.

Liu et al. [101] also examined hepatic and fecal cholesterol, finding a decrease and increase, respectively, with *Ligustrum robustum*. Bile acids and SCFAs measurements revealed an increase and no change, respectively. Altogether, these changes suggest *Ligustrum robustum* decreased total bile acid pool, blunting cholesterol absorption, and increasing cholesterol excretion. mRNA levels of key markers with treatments confirmed this hypothesis. With *Ligustrum robustum*, there was a decrease in NPC1-like intracellular cholesterol transporter 1 (NPC1L1) and an increase in ATP-binding cassette subfamily G Member 8 (ABCG8) in the ileum. Both of these proteins are found in intestinal epithelial cells and transport cholesterol across the cellular plasma membrane. NPC1L1 mediates uptake of intestinal cholesterol into the cell, while ABCG8 effluxes cholesterol from enterocytes into the intestine [110]. In addition, supplementation increased hepatic scavenger receptor class B type 1 (SR-B1), which is a key receptor that absorbs cholesterol from circulation into the liver.

While Liu et al. [101] used 16S rRNA to examine many changes in the gut microbiome, Liao et al. [76] only examined one bacterial genus, *Bifidobacterium*, with RT-qPCR. Liao et al. found that gut levels of *Bifodobacterium* were increased with tea polyphenols. Decreases in the relative abundance of *Lachnospiraceae_FCS020_group, Odoribacter* and *Oscillibacter* were noted with *Ligustrum robustum*, but F/B ratio and alpha-diversity were unchanged.

#### 5.5.3. Other

There are five papers [85,93,104,111,112] in this category of traditional Chinese medicine that are either specific plants/herbs/roots or a combination of these not falling into the above categories. All these studies used ApoE^−/−^ mice and found a reduction in atherosclerosis with treatment in correlation with gut microbiome changes.

The study by Zhang et al. [85] used an herbal medicine that contained berberine as a main active ingredient along with salvianolic acid B. This was called Dingxin Recipe IV and has been used to treat CVD for 2000 years. This study used eight-week-old male mice (*n* = 8/group) and the treatment groups received 12 weeks HFD (21% fat + 0.15% cholesterol) then 12 additional weeks HFD without or with increasing amounts of Dingxin Recipe IV (1.8, 0.9, or 0.45 g/kg/d). Dingxin Recipe IV alleviated HFD-induced weight and fat gain. Aortic sinus cross sections were assessed for plaque with Oil Red O and Masson Trichrome staining and circulating lipids were analyzed. Dingxin Recipe IV prevented atherosclerosis progression and increased collagen fibers indicating enhanced plaque stability. Dingxin Recipe IV also reduced TC, TG, and LDL. Measures of oxidative stress (MDA and lactate dehydrogenase, LDH) in the blood were found to be attenuated with treatment. The endogenous antioxidant SOD was also measured from blood samples and was found to be heightened with Dingxin Recipe IV. As far as gut microbiome changes examined by 16S rDNA sequencing, Dingxin Recipe IV reversed the HFD-induced reduction in diversity. Treatment also decreased the F/B ratio, increased the abundance of *Muribaculaceae, Ruminococcaceae,* and decreased the abundance of *Erysipelotrichaceae, Ileibacterium* and *Allobaculum. Ruminococcaceae* is a known group of butyrate-producing bacteria, and butyrate was found to be heightened in feces of mice supplemented with Dingxin Recipe IV along with propionate. Interestingly, acetate was decreased with treatment. The decrease in *Erysipelotrichaceae* may be beneficial as this bacterium has been shown to increase TMAO production resulting in cholesterol accumulation. Levels of Liver X Receptor (LXR-α) and sterol regulatory element-binding protein 1 (SREBP1); involved in fatty acid metabolism, were measured and found to be decreased with Dingxin Recipe IV. Overall, this herbal medicine reduces atherosclerosis and oxidative stress, as well as modulating the microbiome and lipid metabolism.

In another study by Zhang et al. [93], eight-week-old male mice (*n* = eight/group) were treated with Ophiopogonin D, which is a compound extracted from the *Ophiopogon japonicus* plant known to exert anti-inflammatory and antioxidant effects. Mice were fed HFD (21% fat + 0.15% cholesterol) alone, with either Ophiopogonin D (0.5 mg/kg/d) or simvastatin (5 mg/kg/d) delivered once per day by gavage for 12 weeks. Ophiopogonin D significantly reversed HFD-induced lipid accumulation and aortic intimal thickening and cap thinning, which was assessed in aortic root cross sections stained with Oil Red O and H&E. Masson Trichrome stain was also performed but findings were not reported. Ophiopogonin D also lowered TC, TG, LDL, but not HDL. Blood markers in mice treated with Ophiopogonin D indicated reduced systemic oxidative stress. This included decreases in MDA and LDH and an increase in SOD. In addition, liver markers of inflammation were decreased with treatment, including aspartate aminotransferase (AST) and alanine transaminase (ALT), and liver cryosections stained with Oil Red O found that treatment reversed HFD-induced steatosis. CVD and diabetes are common comorbidities [113]. Interestingly, this study examined glucose tolerance and insulin resistance which they found was improved with Ophiopogonin D, suggestive of reduced risk for T2D. Liver samples were analyzed by RT-qPCR and Western blotting for key markers including mammalian target of rapamycin (mTOR), SREBP1, and stearoyl-CoA desaturase 1 (SCD1) which were found upregulated by HFD alone but reduced with HFD plus Ophiopogonin D treatment. Human fetal hepatocytes (LO_2_ cells) were treated with oleate to induce lipid accumulation and test the effects of Ophiopogonin D in vitro. Results indicated that Ophiopogonin D inhibited phosphorylation of mTOR. In combination with in vivo results, Ophiopogonin D may improve lipid metabolism by the mTOR/SREBP1/SCD1 pathway. Lastly, gut microbiome changes were examined by 16S rDNA sequencing. Both the HFD-induced reduction in diversity and the HFD-induced upregulation of the F/B ratio were reversed with treatment. Furthermore, with the addition of Ophiopogonin D, the relative abundance of *Erysipelotrichaceae* was decreased and relative abundance of *Muribaculaceae* was markedly increased. For genus level changes, Ophiopogonin D increased the relative abundance of *Faecalibaculum* and decreased the relative abundance of *Ileibacterium.* In addition, many fecal metabolites were measured. Ophiopogonin D increased leucine, acetate, ribose, propionate, valine, methionine, glutamate, and lysine while butyrate was unchanged.

There were a number of similar findings between the studies examining Dingxin Recipe IV and Ophiopogonin D and the same researchers carried out these studies. Similarities including lowered atherosclerosis, circulating lipids, oxidative stress indices, SREBP1, the F/B ratio, *Erysipelotrichaceae* and *Ileibacterium* and increased gut diversity, *Muribaculaceae* and propionate were observed with both treatments.

Another Chinese plant that was investigated by Gao et al. [104] was Gypenoside XLIX, which is a main component of the herb *Gynostemma pentaphyllum* (Thunb.) Makino. In general, Gypenosides has been found to have many beneficial pharmacological effects including anti-inflammatory, anti-cancer, anti-atherogenic, neuroprotective, lipid regulatory and hepatoprotective. The age and sex of the ApoE^−/−^ mice were not disclosed, but each group contained 8 mice. A high-fat choline diet (HFCD; 0.15% cholesterol, 21% fat, and 1% choline chloride) was utilized with and without Gypenoside XLIX (30 mg/kg/day by gavage) for 6 weeks. Aortic root cross-sections were stained with Oil Red O showing that HFCD-induced lipid accumulation was attenuated with Gypenoside XLIX treatment. Circulating lipids including TC, TG, LDL were also reduced with the addition of Gypenoside XLIX, while HDL was increased. Plasma TMAO was increased with HFCD and decreased with Gypenoside XLIX while FMO3 was unchanged indicating TMA (produced by gut bacteria) was responsible for the increase in TMAO. Many gut microbiota changes were noted including a surprising increase in the F/B ratio and an increase in the alpha-diversity with treatment. Additional changes in bacterial abundances with Gypenoside XLIX included decreases in TMAO-producers including *Clostridioides* and *Desulfovibrionaceae* and increases in butyrate-producers including *Eubacterium, Roseburia, Bifidobacterium, Lactobacillus,* and *Prevotella*. Gypenoside XLIX also increased fecal SCFAs including acetic acid, propionic acid, and butyric acid. Endogenous antioxidants including SOD and glutathione peroxidase (GSH-Px) were assessed in the serum and liver, respectively. The antioxidants were heightened with the addition of Gypenoside XLIX. On the other hand, liver amounts of MDA, a measure of oxidative stress, were decreased with treatment.

Li et al. [111] assessed the efficacy of 2,3,5,4′-Tetrahydroxy-stilbene-2-O-β-D-glucoside (TSG), which is the main active component of Polygoni Multiflori Radix (PMRP), a root from a plant used in traditional Chinese medicine. TSG and PMRP have been shown to exert beneficial effects including lipid lowering, hepatoprotection, antioxidant, anti-tumor, anti-atherosclerotic but the specific mechanism in atherosclerosis is yet to be elucidated. Male mice (*n* = 7/group) were fed HFD and treated with a water extract of PMRP (1.125 mg/g/day), low dose TSG (0.035 mg/g/day), high dose TSG (0.05 mg/g/day) and simvastatin (0.0025 mg/g/day) for 8 weeks. After treatment, the thoracic portion of the aorta was collected and longitudinally opened and stained with Oil Red O for plaque accumulation, although quantification of plaque area is not provided, the paper states TSG reduced atherosclerotic plaque burden. Serum lipids were analyzed, and both PMRP and TSG reduced TG, but there was no significant effect on TC. PMRP and TSG (high dose) did reduce ox-LDL with the latter also reducing ox-LDL/total serum LDL ratio. This indicates TSG inhibited the oxidation of serum LDL, a crucial step in atherosclerosis progression. TSG combated inflammation by reducing serum molecules including IL-6, TNF-α, VCAM-1, and MCP-1. PMRP also reduced inflammation by down regulating VCAM-1, ICAM-1, and C-C Motif Chemokine Receptor 2 (CCR2). Surprisingly, F/B ratio was increased with the high dose of TSG and PMRP. *Proteobacteria* and *Tenericutes*, which are considered pathogenic, were decreased with low and high doses of TSG. Low and high doses of TSG led to an increase in *Akkermansia* and a decrease in *Helicobacter pylori*. These are two bacteria that are often associated with atherosclerosis which were modulated favorably with treatment. Overall, this study suggests that TSG can reduce lipid accumulation and inflammation and alter the microbiome to reduce atherosclerotic burden.

Wang et al. [112] tested the effects of Qing-Xin-Jie-Yu Granule (QXJYG) in six-week-old male mice (*n* = seven/group). QXJYG is an herbal medicine containing (*Astragalus membranaceus* [Huangqi], *Salvia miltiorrhiza* Bunge [Danshen], *Ligusticum chuanxiong* Hort [Chuanxiong], *Agastache rugosus* [Huoxiang], and *Coptis chinensis* [Huanglian]), and its ingredients have shown efficacy in treating CVD. For this study, two doses (1.664 g/kg/d and 4.992 g/kg/d) were administered via gavage once daily along with HFD (21% saturated fat and 0.15% cholesterol) for 12 weeks. QXJYG reduced HFD-induced increases in serum TC, TG, and LDL. In addition, the high dose of QXJYG increased HDL. QXJYG also blunted inflammation evidenced by reducing serum and aortic mRNA levels of IL-1β and IL-6. Aortic lesions and necrotic core were examined in cross-sections of the aortic root stained with Oil Red O and H&E. The low dose of QXJYG trended toward a decrease in aortic plaque area, but only the high dose was statistically different than HFD alone. QXJYG also showed efficacy in reducing necrotic core size. Immunohistochemical analysis revealed increased smooth muscle cells (SMCs) at surface of plaques, and reduced macrophage and T-cell infiltration in lesions with QXJYG. 16S rRNA analysis showed many changes with a high dose of QXJYG including increased *Roseburia, Aerococcus, Enterobacter, Defluviitaleaceae_UCG_011, Turicibacter, Papillibacter, Jeotgailcoccus,* and *Ruminococcus.* In addition, *Alistipes, Rikenella,* and *Blautia* were less abundant in the QXJYG group. QXJYG also increased hepatic bile acid synthesis enzymes including CYP7A1, and CYP27A1, while decreasing FGF15 and β-Klotho ileal mRNA expression. These changes indicate increased *de novo* bile acid synthesis driving cholesterol excretion. Overall, QXJYG alleviated HFD-induced inflammation, serum lipid accumulation, and plaque burden which was correlated with gut microbiome changes and bile acid metabolism.

### 5.6. Berries

Berries are known to be rich in polyphenols and fiber and while there are many types of this review will focus on two—lingonberries and pomegranate. These berries were studied in atherosclerotic mice in correlation with gut microbiome changes. Interestingly, both berries share a characteristic small round shape and deep red color. Of the four berry studies, two used lingonberries [83,95] and two used pomegranate [77,78]. All studies used six- to nine-week-old ApoE^−/−^ mice (*n* ≥ ten per group) which ranged from four to 10.5 weeks. In addition, three studies [77,78,83] used males, while one used females [95]. Importantly, only the lingonberry studies assessed plaque burden and both studies reported a reduction.

Both lingonberry studies had similar study designs, utilizing HFD and whole lingonberries. The first by Matziouridou et al. [83] only had one experimental intervention, supplementing diet with 44% lingonberries for eight weeks. This study did not see changes in body weight with intervention. The second study by Liu et al. [95] had three experimental interventions, using whole lingonberries (60 g/kg fiber from lingonberry plus 3 g/kg flavonoids), lingonberry flavonoids (60 g/kg cellulose fiber plus 2 g/kg flavonoids), and a lingonberry fiber (60 g/kg) group. The interventions lasted 10.5 weeks and were aimed to delineate whether the polyphenol or fiber was responsible for beneficial plaque-reducing effects. This is a very important question to answer as it has long been known that fiber is beneficial for gut bacteria while the effects of polyphenols on gut bacteria are less widely known.

In contrast, the study designs for the pomegranate studies differed considerably. Rom et al. [77] added acrolein (3 mg/kg/day) with and without pomegranate juice (7 mg gallic acid equivalents—GAE/kg/day) to the drinking water for four weeks. Acrolein is a colorless liquid that forms from burning. It is found in tobacco cigarette smoke, heated saturated and unsaturated fats, and in automobile exhaust. It is also an endogenous byproduct of lipid peroxidation and myeloperoxidase-induced amino acid oxidation in states of oxidative stress and inflammation. This study assessed whether the polyphenols in pomegranate juice could reduce cholesterol, TG, and lipid peroxidases in aortas. Atherosclerosis was not assessed. The second study by Neyrinck et al. [78] used HFD with and without 5% chitin glucan (an insoluble dietary fiber) and with and without 0.5% pomegranate peel extract (40% polyphenols—10% punicalagin and 2% ellagic acid). The interventions were for 8 weeks. This study wanted to determine the role of fiber and polyphenols on the gut microbiome and endothelial dysfunction. In addition, this study reported no changes in body weight and fat mass between HFD alone and with various interventions.

All berry studies examined lipid profile of the mice. For lingonberry treatments, Matziouridou et al. [83] observed a trend towards reduced TC while Liu et al. [95] observed no change. Matziouridou et al. observed a significant decrease in TG, while Liu et al., observed a significant increase in TG (with whole lingonberry and lingonberry fiber but not lingonberry flavonoids). Rom et al. (acrolein and pomegranate juice study) [77] observed a decrease in TC and TG with intervention, while Neyrinck et al. (chitin-glucan and pomegranate study) [78] observed no changes. However, this study did observe a decrease in hepatic TG (chitin-glucan plus pomegranate).

Matziouridou et al. [83] assessed hepatic gene expression of bile acid synthesis genes (CYP7A1, CYP8B1, small heterodimer partner—Shp) finding a significant increase in CYP7A1 with lingonberries. Total levels of cecal SCFAs were also measured and although they were lower in the lingonberry treated mice, propionic acid was higher with treatment.

Liu et al. [95] assessed plasma creatinine, finding a decrease with all lingonberry groups. L-carnitine was assessed and found to decrease in only the whole lingonberry group. TMAO was also assessed and was increased with the flavonoid and fiber groups (but not whole lingonberry group). In addition, betaine and acetyl-carnitine were increased, only in the flavonoid fraction group.

Rom et al. [77] focused on macrophage lipids and oxidative stress. In vivo, they measured aortic lipid peroxidation which was highest in the acrolein group but significantly reduced with pomegranate juice. This study also used in vitro assays to measure ROS, macrophage cholesterol and TG mass and biosynthesis rate, as well as LDL and ox-LDL. This study also assessed macrophage lipid droplets by Oil Red O staining and western blot for key markers of lipid biosynthesis SREBPs, 3-hydroxy-3-methyl-glutaryl-CoA reductase (HMGCR), and diacylglycerol acyltransferase1 (DGAT1). Pomegranate juice reduced the acrolein-induced upregulation of SREBP2, HMGCR, and DGAT1.

Neyrinck et al. [78] assessed inflammation status and mesenteric artery nitric oxide (NO). The pro-inflammatory marker MCP1 was downregulated (chitin-glucan) in adipose tissue and TNF-α, IL-1β and COX-2 were downregulated in the liver (chitin-glucan plus pomegranate). Similar to observations in circulating lipids, no changes were observed in circulating pro-inflammatory and cell adhesion markers (IL-6, IL-10, IL-1β, MIP1α, MCP1, TNF-α, sE-Selectin, sICAM-1, PAI-1, and proMMP-9). Chitin-glucan plus pomegranate also improved HFD-induced endothelial dysfunction. Specifically, the combination treatment increased endothelial NO-synthase in mesenteric arteries and the heme-nitrosylated haemoglobin (Hb-NO) blood levels suggesting that mesenteric arteries have a greater propensity toward NO production.

Lastly, all of the berry studies analyzed the gut microbiome, finding changes between treatments. Interestingly, the two lingonberry studies saw an increase in *Akkermansia* [83,95], while the pomegranate plus chitin glucan saw a decrease [78]. Three studies saw a decrease in the F/B ratio [77,83,95]. Both lingonberry studies [83,95] saw a decrease in alpha-diversity which in general is associated with gut dysbiosis. However, the increase in *Akkermansia* as well as the decrease in F/B ratio seen in both lingonberry studies suggests gut microbiome improvements. Matziouridou et al. [83] saw additional gut changes with lingonberries including heightened genera including *Parabacteroides* and *Clostridium* as well as increases in species including *Blautia producta, Clostridum difficile,* and *Eubacterium dolichum*. In addition, this study saw that lingonberry supplementation decreased total SCFAs but increased propionic acid. Liu et al. observed that whole lingonberry supplementation decreased the abundance of *Oscillospira, Lactobacillus, Mucispirillum* and *Bilophila* while increasing the abundance of *Bifidobacterium*. Other changes were seen with the fiber and flavonoid fractions used in this study, including with flavonoids a decrease in the abundance of *Lactobacillus* and an increase in the abundance of *Bifidobacterium*. With the fiber fraction, a higher abundance of *unclassified S24_7* and *Clostridiales* was observed. Importantly, the whole lingonberry and the flavonoid fraction, but not the fiber fraction, had increased *Akkermansia*.

Additional gut microbiome changes in the chitin-glucan and pomegranate study included a decrease in the HFD-induced increase in *Allstipes* spp. and *Lactobacillus* spp. Similarly, in the pomegranate juice study, treatment reduced acrolein-induced changes. Pomegranate juice decreased *Lachnospiraceae* (specifically, *Coprococcus*) and the *Dehalobacteriaceae* family (specifically, *Dehalobacterium*) and increased *Lactobacillaceae* family (specifically, *Lactobacillus*).

### 5.7. Grains

There are four studies [80,86,90,114] investigating the effects of grains or components derived from grains on atherosclerosis and gut health. All of these studies use male mice; three used ApoE^−/−^ mice [86,90,114] and one used LDLR^−/−^ mice [80]. None of these studies reported data on body weight of the mice.

Two of these studies use millet polyphenols or millet protein [86,90] in four- to five-week-old ApoE^−/−^ mice (*n* = 10/group) for 16 weeks. Millet shell contains polyphenols that exert anti-inflammatory, antioxidant, and lipid-reducing effects. Liu et al. assessed high and low doses of millet shell polyphenols (100 mg/kg, 50 mg/kg) in water along with a HFD. There were additional HFD groups with and without atorvastatin calcium (10 mg/kg) in water. In vitro work performed in human aortic smooth muscle cells (HASMCs) suggested that millet shell polyphenols blunted cell migration, a process crucial to lesion formation. In vivo analysis of atherosclerotic burden was assessed in the whole aorta and aortic sinus cross sections stained with Oil Red O. Similar to the statin, low and high doses of millet shell polyphenols reduced plaque area. Inflammation status was assessed by analyzing blood biomarkers including LPS, TNF-α, and IL-1β, all of which were reduced by millet shell polyphenols. TNF-α, and IL-1β were also assessed by immunochemistry in aortas and were found to be reduced by millet shell polyphenols. As bacterial LPS, a marker of leaky gut, was reduced with polyphenols, tight junction protein status was assessed. Colon mRNA and protein levels of tight junction proteins including occludin, ZO-1, and claudin-1 were increased by millet shell polyphenols in a dose dependent manner. This indicates the polyphenols reduced HFD-induced gut leakage. Next, 16S rRNA gene sequence analysis was performed on the cecal contents of experimental mice. Phyla changes with millet shell polyphenols included an increase in the relative abundance of *Bacteroidetes* and reductions in *Verrucomicrobia* and *Actinobacteria*. Genera changes with millet shell polyphenols included increases in the relative abundances of *Oscillospira* and *Ruminococcus* and a decrease in *Allobaculum*. Overall, millet shell polyphenols were found to remodel the microbiome, changing specific bacteria, in correlation with reduced inflammation, leaky gut, and atherosclerotic burden.

Shan et al. [90] extracted foxtail millet bran protein and applied it along with a high-fat and high cholesterol diet (HFC) in low and high doses (15 and 30 mg/kg, respectively) administered via gavage. This study also utilized a relevant in vitro model. THP-1 cells (a monocyte line) were induced into foam cells by ox-LDL incubation. The foam cells were then treated with millet bran protein hydrolysates, which blunted lipid phagocytosis and secretion of pro-inflammatory molecules (IL-1β and TNF-α). Both this study [90] and the above millet study [86] identified the F4 extracted portion, also referred to as millet shell polyphenols, as the effective component of millet in vitro. Significant reductions were seen in lesion area, assessed in vivo in whole aortas and aortic sinus cross-sections. 16S rRNA sequencing of cecal contents was performed to assess gut microbiome alterations. The HFHC diet reduced diversity and richness and the low dose of millet bran had poor recovery of these indices while the high dose greatly improved these indices. The main phyla change was in the relative abundance of *Firmicutes*, which was significantly increased with millet bran. The treatment also increased the abundance of *Turicibacter* and *Lactobacillus.* Spearman’s analysis revealed that *[Ruminococcus]*, *Allobaculum*, and *Akkermansia* were positively related to inflammation and atherosclerosis. KEGG analysis of 16S rRNA sequencing data revealed pathway upregulations including amino acid metabolism, xenobiotics biodegradation metabolism, and lipid metabolism, while downregulating glycan biosynthesis/metabolism and metabolism of terpenoids and polyketides pathways. These pathways were not further explored in this paper. Lastly, correlation analyses were performed, indicating that the increase in *Lactobacillus* was the most important change with millet bran. Overall, foxtail millet bran protein remodels the gut microbiome to reduce inflammation and atherosclerosis.

Dong et al. [114] investigated the effect of oral supplementation with red yeast rice in seven-week-old male ApoE^−/−^ mice (*n* = six/group) fed HFD for 12 weeks. The whole aorta and aortic cross sections (ascending portion) were stained with Oil Red O for analysis of plaque. Daily oral rice gavage reduced HFD-induced plaque area. Plasma lipids were also assessed, and treatment reduced the HFD-induced increases of TC and LDL, while TG was unchanged. The integrity of arteries and the intestine was examined with transmission electron microscopy. In the arteries, rice treatment inhibited HFD-induced intima thickening and loss of endothelial cells. In the intestine, the HFD-induced loss of integrity and absence of clear structure (tight-junctions and desmosome connections) was improved, and microvilli length restored with rice supplementation. Inflammation status was assessed as atherosclerosis is a pro-inflammatory condition. A component of red yeast rice (monacolin K) has been shown to inhibit HMG-CoA reductase. Therefore, hepatic protein levels of HMG-CoA reductase were assessed, and rice was shown to decrease these levels. Further, tight-junction proteins (junctional adhesion molecule-1, JAM-1 and occludin) were also increased with dietary rice addition. Inflammatory marker protein levels in the intestine were assessed. These included TNF-α and IL-1β as in the two other grain papers. TNF-α and IL-1β were both decreased with rice addition. Additional inflammatory markers involved in toll-like receptor (TLR) cell-signaling cascades were assessed in aortic tissues. These included TLR2, TLR4, and Mitogen-activated protein kinase (MAPK) and according to the figure, rice supplementation decreased all these markers which were increased with HFD alone. 16S rRNA sequencing was performed using cecal samples from mice. Rice supplementation decreased *Firmicutes* independent of a change in *Bacteroidetes*. Other changes with supplementation included an increase in *Bacteroidaceae* abundance and a decline in *Rikenellaceae* abundance. Further analysis of changes discovered that the major significant changes with the addition of rice to the diet included higher abundances of *Bacteroides* and *Anaeroplasma* and lower abundances of *Alistipes, Barnesiella,* and *Flavonifractor*. Lastly, a negative correlation existed between rice supplementation and plasma LDL levels. Overall, red yeast rice led to reductions in inflammatory signaling pathways and significant beneficial changes in both the intestine and aorta and circulating lipids that were associated with gut microbiome alterations compared to HFD alone.

The last paper in this category assessed the efficacy of 14-week addition of dietary oat fiber (0.8%) to HFC diet (46% fat) in male LDLR^−/−^ mice (*n* = 10/group) [80]. As with the red yeast rice, lesion area and intestine improvements were noted; however, changes in the gut microbiota itself were not reported. Both the whole aorta and aortic root cross-sections were stained with Oil Red O for lesion area observations, but only the latter was quantified. Oat fiber significantly reduced plaque area as well as relevant blood markers including blood glucose, insulin, and insulin resistance. Ileum tissue was stained with H&E to assess structural changes. Oat fiber reduced the HFC-induced detrimental changes including damage to the villi, and inflammatory infiltration. These changes indicate improved gut barrier function meaning less permeability and leaking of bacteria into the body. Reduced gut inflammation and improved gut barrier function are known to delay atherosclerosis progression. The effect of oat fiber on gut microbiota-derived metabolites was assessed. With treatment, increases were seen in L-tyrosine and niacinamide concentrations, while decreases were seen in isobutyrylcarnitine, valerylcarnitine, 1-methylguanosine, and 2- methylguanosine concentrations. Protein amounts of pro-inflammatory signaling molecules in both the TLR4/NF-κB signaling cascade and the NLRP3 inflammasome pathway were assessed in aortic and colon tissues from mice. Oat fiber decreased TLR4, myeloid differentiation primary response 88 (MyD88), TIR-domain-containing adapter-inducing interferon-β (TRIF), NF-κB p65, NLRP3, apoptosis-associated speck-like protein containing a CARD (ASC), and IL-1β in aortic and intestinal (distal colon) tissues. IL-18 was also decreased in the colonic tissue. In the aortic tissues alone, the G-couple protein receptor 109A (GPR109A) was decreased with oat fiber. In the colon tissues alone from mice treated with oat fiber, caspase-1 was decreased and ZO-1, and occludin were increased. Overall, oat fiber supplementation blunted atherosclerotic lesion formation in part by changing gut microbiota-derived metabolites and by reducing intestinal inflammation and improving gut barrier integrity.

### 5.8. Other Interventions

There are two studies [84,92] that suggested phytochemical rich foods/extracts alleviated atherosclerotic burden in correlation with changes to the host gut microbiota. One study used six-week-old male LDLR^−/−^ mice (*n* = 12/group) [84] and one used seven-week-old ApoE^−/−^ male mice (*n* = 8/group) [92]. The first study by Gao et al. [84] examined the effects of a fruit and vegetable mix (F&V mix), which was added at 15% as a freeze-dried powder made from the 24 most widely consumed fruits and vegetables in the U.S. The amount delivered to the mice per day was equivalent to eight to nine servings for humans and was given to the mice for 20 weeks. Aortic plaque was assessed in the whole aorta using the Oil Red O staining protocol. Mice were fed an atherogenic diet (27% kcal fat, 0.55 g/kg cholesterol) with and without F&V mix. F&V mix significantly reduced diet-induced atherosclerosis. F&V mix however did not affect the body weight of the mice. Furthermore, hepatic steatosis, serum TG and VLDL were reduced, while HDL was upregulated with the addition of F&V mix. TC and LDL were not significantly changed although they trended downward compared to the atherogenic diet. TNF-α was also assessed in the serum and liver and interestingly F&V mix reduced serum values while increasing liver protein values. In the liver, mRNA levels of TNF-α and fatty acid synthase (FASN) were reduced with the addition of F&V mix. Gut microbiome changes were also observed including an increase in diversity with intervention. On the phyla level, the relative abundance of *Verrumicrobia* was greatly decreased, while *Firmicutes*, *Bacteroides,* and *Actinobacteria* relative abundances were increased. The relative abundances of *Leuconostoc, Trichococcus, Turicibacter*, and *Dorea* were increased with the addition of F&V mix. In addition, two bacteria that are considered beneficial (*S24–7* and *Clostridiales*) were negatively correlated with aortic plaque, liver steatosis, and liver FASN mRNA levels. Overall, this study suggests that consuming a large and diverse number of fruits and vegetables reduces atherosclerosis and hepatic steatosis in mice in correlation with improved dyslipidemia, gut microbiota alterations, and dampened inflammation.

Dong Liu et al. [92] investigated the potential effects of an astaxanthin-rich extract on mitigating plaque burden and gut microbiome dysbiosis. Astaxanthin is a carotenoid with a red pigment present in algae and salmon and synthesized by a number of bacteria, microalgae, and yeast. It has been investigated in a number of studies for skin health due to its photoprotective, anti-inflammatory and antioxidant effects [115]. HFD was applied to induce atherosclerosis with and without two different doses of astaxanthin (164, 329 mg/kg diet). There was an additional HFD plus atorvastatin (65 mg/kg diet) group. The extract reduced HFD-induced body weight gain. Astaxanthin reduced HFD-induced elevations of TC, TG, non-HDL cholesterol, and glucose, while increasing HDL in serum. The high dose of astaxanthin alleviated hepatic TC and TG which were elevated with HFD. Interestingly, retinal fundus large blood vessels, which are present on the interior lining of the eyeball, were assessed as these are known to exhibit distinct morphology in ApoE^−/−^ mice similar to changes in diabetic retinopathy. The vascular lesions were improved by astaxanthin. Immunohistochemistry of retinal CD31 showed that astaxanthin reduced micro retinal density of superficial retinal microvessels vs. HFD. Aortic lesions were assessed with Oil Red O staining of the whole aorta and astaxanthin conferred significant reductions. mRNA levels of key markers in the liver, jejunum, and colon were assessed. Astaxanthin increased AMPKα, LXR-α, and CYP7A1 mRNA levels in the liver. In addition, liver levels of FXR were increased with the high dose of astaxanthin. Additional changes suggested alterations in cholesterol metabolism. Specifically, astaxanthin increased jejunum gene expression of ABCG5/8 while reducing NPC1L1, acetyl-CoA acetyltransferase 2 (ACTA2) and microsomal triglyceride transfer protein (MTTP). Astaxanthin also appeared to improve gut barrier function by increasing colonic gene expression of junctional adhesion molecule-A (JAM-A), occludin, and mucin-2. Fecal sterols were also assessed and were found to be elevated when astaxanthin was applied vs. HFD alone. Specifically, coprostanol and campesterol were increased. All acidic sterols which included chenodeoxycholic acid (CDCA), lithocholic acid (LCA), deoxycholic acid (DCA), and cholic acid were also greatly increased with the addition of astaxanthin. This data suggests astaxanthin supplementation increases fecal excretion of sterols/bile acid compounds. Gut microbiome changes were also noted with the addition of astaxanthin. At the phylum level, *Verrucomicrobia* and the F/B ratio were increased with astaxanthin compared to HFD alone. Addition of astaxanthin increased the relative abundances of *Akkermansia, Bacteroides, Oscillibacter, and Ruminiclostridun_9* and decreases in the relative abundances of *Alloprevotella, Desulfovibrio, Muribaculum, Odoribacter,* and *Parabacteroides.* Additional analysis (LDA score) suggested the greatest change was in the *Akkermansia* genus in the *Verrucomicrobia* phylum. Overall, this study suggests that astaxanthin is effective at reducing atherosclerosis macro- and microvascularly and promoting bile acid excretion and growth of *Akkermansia* in mice.

### 5.9. Interventions in ApoE^−/−^ Mice That Do Not Reduce Plaque

There were three studies in which supplementation failed to significantly reduce plaque. The first study treated eight-month-old male ApoE^−/−^ mice with the purified AIN-93g (growth diet) supplemented with and without Brussels chicory for 20 weeks [81]. In this study, the intervention did not significantly alter the body weight of the mice. The other study did not disclose sex or age of mice but treated ApoE^−/−^ on an atherogenic diet (42% kcals from fat) with and without green coffee extract (administered by gavage) for 14 weeks [82]. In contrast, this study found the extract reduced the effects of HFD’s increase on body weight. The last study investigated whole brown bean and its fiber along with HFD for 10.5 weeks in six-week-old ApoE^−/−^ female mice [97].

The Brussels chicory (also known as Belgian endive) supplemented into the diet at 0.5% (freeze dried) was chosen as it is common in the Mediterranean diet. Other Mediterranean foods such as nuts and olive oil (which are also rich in polyphenols) are known to play a role in reducing CVD [116]. The green coffee extract utilized in the second study was reported to contain 220 mg/kg chlorogenic acid equivalents. Chlorogenic acids are the most abundant phenolic compounds. While overall plaque volume, which was assessed through cross sections stained with H&E, and plasms lipids did not change with Brussels chicory, other plaque features did. Most noteworthy is the significant increase in plaque stability with Brussels chicory which was observed in the cross sections (H&E staining and Sirius Red—staining for collagen). Necrotic core size was reduced while fibrous cap and collagen thickness was increased. In addition, favorable gut changes were noted with Brussels chicory including a decrease in intestinal permeability and an increase in *Ruminococcaceae* abundance, which produces beneficial SCFAs. Other positive changes included reduced fecal and serum LPS concentration as well as reduced serum pro-inflammatory markers IL-1β and TNF-α.

Likewise, green coffee extract induced favorable changes independent of plaque and plasma lipid reductions. Metabolic changes were noted including improved blood glucose, insulin resistance, reduced weight and fat gain, lower inflammatory infiltrate in fat tissue and protection against liver damage (assessed by histology). Green coffee extract also increased liver IL-6, an interleukin that can function as pro- or anti- inflammatory depending on the context [117]. Gut microbiome changed with green coffee extract included increases in abundance of bacteria from operational taxonomic units (OTUs) including *Mogibacteriaceae* (negatively associated with risk of thrombosis), *Coprococcus, Dorea, Ruminococcus, Firmicutes*, and *Desulfovibrio* (anti-inflammatory effects and linked to liver detoxification). OTUs are widely accepted to describe bacterial communities and are defined as a collection of 16S rRNA sequences that contain a certain percentage of sequence divergence.

Jiyun Liu et al. [97] examined the effects of whole brown bean and its isolated fiber fraction on atherosclerosis and the gut microbiota. Mice (*n* = 10/group) were fed HFD to induce plaque accumulation. Either whole bean or bean fiber, each containing 60 g/kg cellulose or dietary fiber from beans, was added to HFD and applied to two additional groups of mice for 10.5 weeks. The reduction in plaque, assessed in aortic root cross sections, was nonsignificant but trended in the right direction for both whole bean and bean fiber (*p* ≤ 0.1). Plasma lipids were largely unchanged by treatments. Cecal SCFAs were assessed and both the whole bean and bean fiber diets increased total cecal pool. Specifically, acetic acid, propionic acid, butyric acid, and minor acids were increased with both bean diets. Plasma methylamines were assessed and surprisingly TMAO was increased by both bean diets compared to HFD alone. Additional changes were seen in mice supplemented with the bean fiber diet including decreased choline and creatine vs. HFD. Gut microbiome changes were assessed, and the whole bean group had higher alpha-diversity than the HFD group. Both bean groups had elevated abundances of phyla *Actinobacteria* and *Bacteroidetes* and a lower F/B ratio vs. the HFD group. Genus level changes with both bean diets included a greater relative abundance of unclassified *S24-7, Prevotella, Bifidobacterium*, and unclassified *Clostridiales*, and a lower relative abundance of *Lactobacillus*. Although plaque data was insignificant, positive effects in the trend toward decrease in atherosclerosis and positive influence on gut microbiome health were seen with both bean diets.

Overall, while atherosclerosis was not reduced by polyphenol rich Brussels chicory, green coffee extract, or brown bean, the treatments improved health of the mice. Brussels chicory promoted a more stable plaque phenotype, reduced systemic inflammation, and increased abundance of butyrate-producing bacteria. Green coffee extract also modulated the gut microbiome and improved liver health and inflammation indices while lowering weight and fat gain in mice. Lastly, brown bean supplementation trended toward decreasing atherosclerotic plaque and improved gut microbiota and enhanced SCFA production.

## 6. Discussion

**Figure 4:**
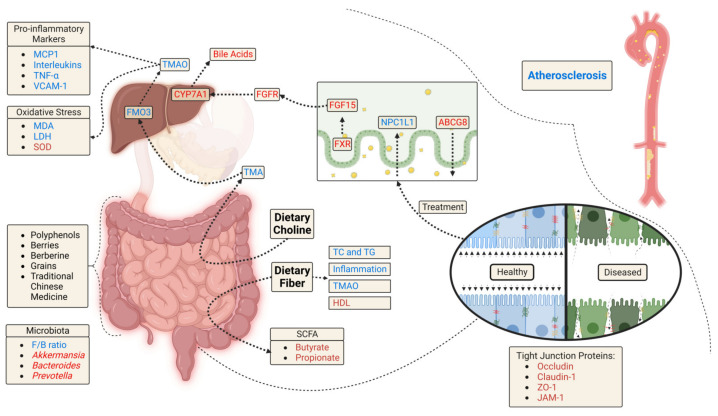
Mechanisms regulating plaque by the microbiome. Nutritional interventions reduced plaque in the aorta of mice by modulating the microbiome. The F/B ratio was reduced while the relative abundance of *Akkermansia*, *Bacteroides,* and *Prevotella* was upregulated. Changes in the microbiome were associated with increases in SCFAs levels and bile acid excretion in the liver, likely mediated by upregulation of CYP7A1. Lower levels of TMA and TMAO also correlated with reduced inflammation and oxidative stress in circulation and with reduced gut barrier permeability. Red signifies upregulation and blue downregulation in expression mediated by nutritional interventions. Created with BioRender.com (accessed on 8 February 2023).

The mechanism by which the nutritional interventions described here reduced plaque is summarized in Figure 4. The overall mechanism involved reduced inflammation, oxidative stress, increased production of SCFAs, and improvements in intestinal barrier permeability. Reduced microbiome dybiosis and TMAO levels and increased bile acid excretion due to increased expression of CYP7A1. Each of these mechanisms is discussed in detail in the following sections and summarized in Supplementary Table S1.

### 6.1. Analysis of Plaque

To summarize, almost all the included studies assessed plaque except the two studies supplementing mice with pomegranate. Three studies did not see reductions in plaque with treatment (Brussels chicory, green coffee extract, and whole brown bean) and two studies, supplementing with procyanidin A2 and PMRP/TSG, did not include quantitative data for plaque. It is imperative to note that plaque was assessed by different methods in different papers. Some assessed plaque by analyzing the whole aorta, including the arch and descending portion, while others assessed cross sections of the aortic root. Most of the studies and all that examined aortic root cross sections utilized oil red O to stain the plaque for quantification. Frequently an H&E counterstain was performed but often quantification or description of the data was not provided. Examination of the aorta by *en face* method on a black max background, the method our lab utilizes, does not require staining as the plaque is bright white [75]. A major drawback of analysis of only the aortic root is that it can miss plaque accumulation in other portions of the aorta. Although plaque does preferentially accumulate in the aortic arch and branches due to turbulent blood flow related to the structure of this portion, certain CVDs such as aortic aneurysms, are characterized by plaque burden in the descending portion of the aorta. In addition, our lab has found very interesting sex-dependent and treatment dependent changes in the arch versus descending aortic portions. These changes included HFD-induced plaque reduction in only the aortic arch with blackberry (2%) [118], but plaque reduction in the arch and descending aorta with gallic acid (0.2%) [75], a major polyphenol in blackberry, in male mice. No changes in plaque burden were seen in female mice. This brings us to an important point about the studies, which is that often either males or females are used, instead of a concurrent use of both. Our gallic acid study and a study supplementing with tea polyphenols were the only two out of the ones reviewed here that used both male and female mice. While we reported sex differences, the study utilizing tea polyphenols did not report sex differences, which is a major limitation.

Four studies including those treating with berberine [88], Ginkgolide B, Dingxin Recipe IV, and Ophiopogonin D analyzed collagen deposition in the plaque through Masson’s trichrome staining. Only two of these studies quantified data. Results from this stain were very informative as Dingxin Recipe IV was observed to increase collagen, which enhances plaque stability. In contrast, berberine [88] was observed to decrease collagen in the plaque. While this finding is not elaborated upon, it could be due to the overall reduction in aortic morphological changes (i.e., less atherosclerotic burden) observed with treatment.

### 6.2. Blood Lipids, Glucose, and Insulin Resistance

There are many changes observed across multiple interventions. As CVD and atherosclerosis are associated with perturbations in glucose and lipid profile in humans, many papers analyzed the blood for glucose, TG, and cholesterol including TC, VLDL, LDL, and HDL. HDL is known to carry cholesterol from the blood to the liver, thus is considered atheroprotective. As T2D is a risk factor for CVD, glucose is a relevant marker.

A significant reduction in circulating TC was observed in twelve studies including those applying curcumin [105], naringin, Ginkgolide B, *Gingko biloba*, tea polyphenols, Dingxin Recipe IV, Ophiopogonin D, Gypenoside XLIX, QXJYG, pomegranate juice, red yeast rice, and astaxanthin-rich extract. Fourteen studies saw significant decreases in TG including those supplementing curcumin [105], procyanidin A2, Ginkgolide B, *Gingko biloba*, Dingxin Recipe IV, Ophiopogonin D, Gypenoside XLIX, TSG, PMRP, QXJYG, lingonberry, pomegranate juice, F&V mix, and astaxanthin-rich extract. Interestingly, while one study observed a decrease in TG with lingonberry [83], the other lingonberry study observed an increase in TG [95]. Overall, many of the studies observed decreases in both TC and TG. Three studies including those treating mice with naringin, tea polyphenols, and red yeast rice, only observed a decrease in TC. While four studies including those supplementing mice with procyanidin A2, TSG, PMRP, and F&V mix only observed a decrease in TG. Interestingly, while all four berry studies measured circulating TC and TG, only pomegranate juice observed significant decreases in both markers.

Eleven studies that observed significant reductions in circulating LDL were reported with curcumin [105], procyanidin A2, naringin, Ginkgolide B, *Gingko biloba*, tea polyphenols, Dingxin Recipe IV, Ophiopogonin D, Gypenoside XLIX, QXJYG, and red yeast rice. Astaxanthin-rich extract attenuated non-HDL cholesterol, but the specific lipoproteins were not reported. Only a few studies reported reductions in VLDL with intervention, including those supplementing Ginkgolide B and F&V mix. An increase in atheroprotective HDL was observed in seven studies, including those supplementing with curcumin, procyanidin A2, Ginkgolide B, Gypenoside XLIX, QXJYG, F&V mix, and astaxanthin-rich extract. In contrast, a decrease in HDL, which was partially explained by total cholesterol decrease, was observed with tea polyphenols.

Four studies observed circulating glucose reductions with treatment, including those supplementing Ginkgolide B, astaxanthin-rich extract, oat fiber, and green coffee extract. Rather than measuring blood glucose upon sacrifice, the Ophiopogonin D study examined glucose sensitivity with an oral glucose tolerance test, seeing improvements with treatment. In addition, three studies saw improvements in insulin resistance including those treating mice with Ophiopogonin D, oat fiber, and green coffee extract. Insulin resistance is important in relation to risk for T2D, a common comorbidity of CVD. It is important to note that the two former treatments were associated with reduced plaque burden while green coffee extract was not associated with reduced plaque burden. All three studies saw gut microbiome changes. Thus, the reduction in plaque by intervention is not always associated with improvements in the lipid profile.

### 6.3. Gut Microbiome Changes

Gut microbiota was assessed in all papers except the one supplementing mice with oat fiber and the excluded curcumin paper [79]. The F/B ratio indicates intestinal homeostasis and absence of gut dysbiosis. The decrease in F/B ratio was seen across numerous papers reviewed including those treating mice with quercetin, resveratrol, geraniin, curcumin, procyanidin A2, gallic acid, *Ginkgo biloba* and Ginkgolide B, Dingxin Recipe IV, Ophiopogonin D, pomegranate juice, lignonberries, and brown bean [75,77,83,85,89,91,93,94,95,96,97,98,103,105]. The decrease in F/B ratio is often associated with reduced obesity in humans; however, a meta-analysis observed instances of the opposite trend [119]. Several papers did report an increase in the F/B ratio with treatments including naringin, berberine, Gypenoside XLIX, TSG (high dose) and PMRP, and astaxanthin-rich extract [88,92,102,104,111]. This could indicate an improvement of intestinal inflammatory response. In humans, a decreased F/B ratio is associated with inflammatory bowel conditions, including Crohn’s Disease and ulcerative colitis [119]. Overall, examination of bacteria besides the major phyla appears to be crucial in unraveling whether treatment exerts beneficial or harmful effects on the host microbiome.

Several bacterial genera that have been identified as beneficial including *Akkermansia, Prevotella,* and *Bacteroides* were changed by multiple interventions. An increase in *Akkermansia* was observed with thirteen interventions including, gallic acid (males), quercetin, resveratrol, geraniin, curcumin, procyanidin A2, berberine, *Gingko biloba*, TSG, lingonberry (both), millet bran, and astaxanthin-rich extract. Additionally, in our gallic acid study, the lack of effect in plaque seen in females correlated with a reduction in *Akkermansia.* Important to note is the geraniin study in mice wherein geraniin plus antibiotics increased *Akkermansia*, indicating this polyphenol was directly responsible for the elevated bacterial abundance.

The correlation between increased *Akkermansia* and polyphenol consumption has been observed previously in humans, as have correlations between increases in *Prevotella* and *Bacteroides* with polyphenol consumption [43]. In this review, *Prevotella* was increased in mice treated with Gypenoside XLIX [104] and whole brown bean and its fiber [97] and decreased in mice treated with resveratrol [96]. In addition, the family to which genus *Prevotella* belongs, *Prevotellaceae,* was increased with procyanidin A2 [89]. Another member of this family, *Alloprevotella* was increased with geraniin [98] and *Gingko biloba* [94] and decreased with astaxanthin-rich extract [92]. *Prevotellaceae* are butyrate producers. In colonic macrophages treated with *Prevotellaceae*, butyrate was shown to suppress inflammation by activating PPARα and downregulating NF-κB-induced IL-1β and TNF-α upregulation [120]. *Bacteroides* was increased with a number of treatments in mice including quercetin, resveratrol, geraniin, berberine, Ginkgolide B, red yeast rice, F&V mix, and astaxanthin-rich extract [84,91,92,96,98,100,103,114], while reductions were observed with naringin [102]. Overall, the largest reported correlation between plaque-reducing treatments and genera changes was with *Bacteroides* as eight studies observed increases in abundance.

### 6.4. Gut Barrier Function

In addition to changes in the gut microbiota, intestinal proteins indicative of a healthy gut was assessed. These included colonic tight-junction proteins including occludin, claudin-1, ZO-1, JAM-1, and JAM-A. Millet shell polyphenols, red yeast rice, oat fiber, and astaxanthin-rich extract increased occludin. *Ginkgo biloba* and millet shell polyphenols increased claudin-1 and ZO-1. Oat fiber also increased ZO-1. JAM-1 was increased with red yeast rice while JAM-A was increased with astaxanthin-rich extract. Mucin-2, which is a major component of the mucus skeleton, providing gut lubrication and protection, was increased by astaxanthin-rich extract. It is interesting that as a group, grains promote these changes, however many studies simply did not assess these markers. Overall, these changes indicate a healthy gut barrier is promoted with certain treatments.

### 6.5. Bile Acid and Lipid Metabolism

Bile acids are the product of cholesterol metabolism, produced in the liver and secreted into the small intestine where they aid in fat digestion and absorption [121]. These acids were assessed in a number of studies. Quercetin, resveratrol, naringin, astaxanthin-rich extract, and *Ligustrum robustum* addition in the diet appear to increase bile acid excretion. This indicates that the interventions facilitate cholesterol elimination from the mice, decreasing plaque accumulation. *Ginkgo biloba* partially restored serum secondary bile acids, which were decreased with Western diet [94]. While increased total bile acid excretion is considered a good indicator of lipid metabolism and elimination, a decrease in secondary bile acids may be a risk factor for atherosclerosis. A particularly toxic secondary bile acid, LCA, has been identified as decreased in serum and fecal samples in humans with coronary heart disease [122,123].

As stated, cholesterol is removed from the body after conversion to bile acids in the liver. A number of CYP enzymes (CYP7A1, CYP7B1, CYP27A1, CYP8B1) are responsible for cholesterol catabolism and bile acid biosynthesis [124] and were evaluated in papers reviewed here. Procyanidin A2, naringin, QXJYG, lingonberry, and astaxanthin-rich extract [83,89,92,102,112] increased expression of various CYP enzymes. This indicates an elevation in cholesterol elimination which is beneficial for reducing atherosclerosis.

Three studies including those treating mice with resveratrol, naringin, and astaxanthin-rich extract [92,96,102], examined the expression of key markers in the enterohepatic FXR-FGF15 axis. FGF15 is a key endocrine growth factor of enterocyte origin and is released in response to ileal bile acid absorption. As a receptor activated by bile acids, FXR controls bile acid metabolism. FXR is present in both the intestine and liver. Activation of the FXR-FGF15 axis promotes bile acid synthesis. Resveratrol [96] and astaxanthin-rich extract [92] stimulated the FXR-FGF15 axis, while naringin [102] dampened it. The FXR also plays a role in lipid metabolism. ABC transporters, involved in lipid metabolism, were assessed in the astaxanthin-rich extract study as well as the procyanidin A2 [89], and *Ligustrum robustum* [101] studies. Astaxanthin-rich extract and *Ligustrum robustum* increased the intestinal expression of ABCG5/8, while procyanidin A2 increased the hepatic expression of ABCA1. Both ABC transporters are involved in cholesterol efflux. The elevated ABCG5/8 indicates suppressed cholesterol reabsorption by enterocytes and was evaluated in tandem with intestinal NPC1L1. NPC1L1 mediates cholesterol uptake into the cell and was decreased with both astaxanthin-rich extract and *Ligustrum robustum*. Overall, these results indicate that the treatments promote hypocholesterolemic activity.

### 6.6. Gut Metabolites

Metabolites produced by bacteria in the gut including TMAO and SCFAs were assessed in a number of studies. Briefly, choline and L-carnitine from the diet undergo bacterial fermentation in the gut and TMA is produced. TMA travels to the liver where FMO3 converts it to TMAO. TMAO in circulation is associated with a number of diseases including metabolic syndrome [125], T2D [126] and atherosclerosis/CVD [127]. TMAO was reduced by treatments including resveratrol, geraniin, curcumin (cd), naringin, Ginkgolide B, *Ligustrum robustum*, and Gypenoside XLIX. Surprisingly, the whole brown bean and its isolated fiber as well as lingonberry flavonoid and lingonberry fiber increased TMAO. The conclusion from these studies, in particular the lingonberry study, in which higher TMAO was found is that the animal model (ApoE^−/−^) and sex (female) likely play a role. ApoE^−/−^ mice have been shown to have inverse correlations between plaque and TMAO. In addition, female ApoE^−/−^ mice have been shown to express higher levels of TMA-converting enzymes [128].

In terms of TMAO present in foods, fish have been shown to contain high levels of TMAO, while also containing anti-atherogenic omega-3 fatty acids. Wang et al. [129] measured the levels of TMA/TMAO and the omega-3 fatty acids EPA and DHA in several species of fish and seafood. This study reported that deep-sea fish, like orange roughly, as well as cod, lobster, snow crab, squid and halibut had high levels of TMA/TMAO, while freshwater fish contain very low levels of these molecules. Interestingly, orange roughly, lobster and cod had low levels of EPA and DHA. In contrast, salmon (farm raised), trout, mussels, tuna (canned) and walleye showed the highest levels of EPA/DHA and undetected or very low levels of TMA/TMAO. Importantly, this study also measured circulating TMAO levels after a meal containing seafood or fish with low (shrimp, canned tuna, and salmon) or high (fish sticks) TMA/TMAO content. Overall, TMAO increased in circulation after the meal, but returned to baseline after 24 h. Thus, although the levels of TMA/TMAO varied significantly in seafood and fish, the effects of these increases in the participants are likely less damaging due to the rapid elimination from circulation. Based on this study, the fish with the highest benefits to human health is trout because of its high content of omega-3 fatty acids and almost undetected content of TMA/TMAO.

The liver expression of FMO3 was assessed in a handful of studies. FMO3 was heightened with resveratrol [96] and geraniin [98] and decreased by Ginkgolide B [91]. In addition, FMO3 was unchanged by *Ligustrum robustum* [101] and Gypenoside XLIX [104], suggesting TMA production was responsible for reduced TMAO.

In terms of beneficial production of gut metabolites, many studies saw an increase in various SCFAs with treatment. *Ginkgo biloba*, Dingxin Recipe IV, Gypenoside XLIX, and whole brown bean and its fiber observed increased total SCFAs [85,94,97,104]. The three SCFAs that were increased across multiple studies included acetic acid, propionic acid, and butyric acid. In humans, these three SCFAs are the most abundant, making up 90–95% of the total SCFAs [130]. Notably, while lingonberry did not increase total SCFAs, an increased in propionic acid was observed [83]. SCFAs are taken up by colonocytes and travel through the portal vein to the liver than to other tissues. SCFAs can reduce inflammation, which is important in the context of atherosclerosis, an inflammatory condition. One example of this is a study by Aguilar et al. [131] in which ApoE^−/−^ mice were fed a butyrate-rich diet (1%) for ten weeks. Decreased aortic plaque as well as changes indicating lower macrophage migration and enhanced plaque stability were observed in the mice fed butyrate. These changes were linked to lower NF-κB activation and reduced pro-inflammatory molecule secretion in human endothelial cells.

### 6.7. Inflammation

Many studies assessed blood levels of inflammatory markers. Several pro-inflammatory molecules that were assessed in a handful of studies included TNF-α, IL-6, and IL-1β. Another interleukin inversely associated with atherosclerosis, IL-10, was also assessed.

Quercetin did not change TNF-α or IL-10, but reduced IL-6 [103]. Geraniin also reduced IL-6, as well, while increasing IL-10 [98]. Geraniin, *Ginkgo biloba*, millet shell polyphenols, and Brussels chicory reduced IL-1β and TNF-α [81,86,94,98]. TSG reduced IL-6, while TSG and F&V mix lowered TNF-α [84,111].

The chitin-glucan and pomegranate study [78] assessed all four markers in the blood but saw no change. However, this study did see decreases in TNF-α and IL-1β in the liver. The millet shell polyphenol study [86] assessed TNF-α and IL-1β in aortas, observing reductions with treatment. The red yeast rice paper [114] assessed TNF-α and IL-1β in the intestine seeing decreases. With F&V mix addition, decreased protein of TNF-α, while decreased mRNA levels were observed [114]. Lastly, cells treated with millet bran protein hydrolysates showed reduced TNF-α and IL-1β [90]. Across studies TNF-α and IL-1β are correlated to the reduction in atherosclerosis-associated inflammation. As mentioned previously, butyrate has been shown to activate PPARα and downregulate NF-κB-induced IL-1β and TNF-α increases [120]. This could be a potential mechanism of action for these interventions. NF-κB/p65 was reported as changed in two studies. Oat fiber [80] and curcumin [105] reduced protein amounts in the aorta, while oat fiber alone reduced protein levels in the colon.

Adhesion molecules, including ICAM-1 and VCAM-1 were assessed in several studies. These molecules promote atherosclerosis through enhancing the migration and adhesion of inflammatory cells. Dietary additions including procyanidin A2 [89] and TSG/PMRP [111] reduced these molecules.

### 6.8. Oxidative Stress

Several studies measured indices of oxidative stress. Blood levels of the endogenous antioxidant SOD was increased with dietary addition of Dingxin Recipe IV, Ophiopogonin D, and Gypenoside XLIX [85,93,104], which were all Chinese medicine treatments. In contrast to SOD, heightened blood levels of MDA and LDH are indicative of oxidative stress. MDA is produced through lipid peroxidation of fatty acids thus can be used to assess lipid peroxidation. Levels of MDA in serum [132] and saliva [133,134] are higher in human atherosclerotic patients. Quercetin [103], procyanidin A2 [89], Dingxin Recipe IV, Ophiopogonin D, and Gypenoside XLIX attenuated blood levels of MDA. LDH is an enzyme of anaerobic metabolism, catalyzing the reversible reaction of lactate to pyruvate while reducing NAD+ to NADH. Blood levels of LDH have been shown to correlate with atherosclerosis and CVD risk [135]. Two of the traditional Chinese medicine interventions found that LDH was also attenuated with treatments, including Dingxin Recipe IV and Ophiopogonin D. The Chinese treatments that increased endogenous antioxidant capacity through SOD while simultaneously reducing one or both oxidative stress markers (MDA and LDH), likely conferred more protection against oxidative stress than either alone. Overall, it is disappointing more studies did not examine these markers.

## 7. Conclusions

While there was overlap between several studies in terms of atherosclerotic burden correlating with microbiome alterations, inflammation, oxidative stress, and bile acid and fat metabolism, all of the papers did not measure the same markers. Therefore, it is difficult to conclude the overlap in the exact mechanisms of action. The aim of this review was to provide an updated evaluation of whether and how dietary interventions modulate the gut microbiome to reduce atherosclerosis. It is remarkable that even an atherosclerotic perturbed gut can benefit from specific dietary interventions. The majority of the studies reviewed herein link selected dietary interventions to foster/restore gut microbiome eubiosis (homeostasis) and reduced atherosclerosis, which is an emerging area of research as many of the studies were published within the last two years. However, many of these studies are correlative in nature. Only two studies [89,96] knocked down the host microbiome with antibiotics in tandem with treatment with the notion to provide a mechanistic link between the gut microbiome and dietary interventions reducing plaque. The host gut microbiome can be crucial in breaking down substances, namely polyphenols. The resident gut microbes also have been shown to contribute to atherosclerotic burden, namely by increasing TMAO, making the gut microbiome depletion a bit more complicated of a model. Specifically, antibiotic-induced knockdown of the gut microbiome reduces TMAO-induced plaque burden because TMA cannot be converted to TMAO by gut microbes [136]. Still, knocking out the gut microbiome followed by providing a treatment requiring breakdown by the gut can show both the requirement and symbiotic nature of a dietary intervention with the gut microbiome. Only one study utilized gut microbiome transplantation [94]. Gut transplantation is arguably a better method, showing that the gut microbiome improvements with intervention can indeed transfer a reduced atherosclerosis phenotype to a new host.

Most of the studies reviewed utilized ApoE^−/−^ mice rather than LDLR^−/−^ mice, making comparisons easier as the models have different characteristics which are reviewed in detail elsewhere [21]. On the other hand, the lack of using both male and female mice is a limitation, as only two papers [75,76] used both males and females and one did not compare the two sexes. It is well known that in humans, specific sex-dependent risk factors exist, making it crucial to use both sexes in murine model investigations. Women have a lower risk for CVD at ages under 50, while their risk increases post-menopause with lifetime risk similar to males [137]. In addition, females are more at risk for stroke [138] while males are at a higher risk for heart attack [139]. As mentioned before the lack of fecal transplantation approaches in the selected studies is a major limitation in the field. Additional limitations include (1) inconsistency in the method used to measure plaque (H&E vs. *en face*), (2) the section of the aorta analyzed (aortic sinus, aortic root, arch, descending aorta), and (3) inconsistencies in the measured outcomes as not all studies measured SCFAs, TMAO, and bile acid levels and metabolism.

Overall, although the design (treatment duration, diet composition, age of mice) of the studies varied significantly, almost all showed reduction in plaque with major changes in the gut microbiome. The significance of this systematic review and a Meta-analysis recently published by our group [140] is the identification of *Akkermansia* as a major bacterial genus modulated by nutritional interventions. *Akkermansia muniphila* has shown health promoting effects including boosting immune function [141], reducing kidney fibrosis [142] and improving cognitive function [143]. Thus, the nutritional interventions reviewed here are of potential benefit for patients suffering from these conditions in addition to CVDs. Future research is needed to evaluate the role of this bacterium in human health and to translate the nutritional interventions discussed here to humans to improve microbiome health and to reduce CVD and other chronic inflammatory diseases.

## Data Availability

No new data were created or analyzed in this study. Data sharing is not applicable to this article.

## Supplementary Materials

The following supporting information can be downloaded at: https://www.mdpi.com/article/10.3390/nu15051212/s1, Table S1: Major changes induced by intervention in circulation and tissues.
