# Supplementary material for: The Role of Phytochemicals and Gut Microbiome in Atherosclerosis in Preclinical Mouse Models
Source: Nutrients. 2023 Feb 28;15(5):1212. doi: 10.3390/nu15051212 (PMC10005405; doi:10.3390/nu15051212)
Supplement: Supplementary file 1 [file nutrients-15-01212-s001.zip › nutrients-2219600-supplementary.pdf]

Table S1. Major changes induced by intervention in circulation and in tissues

Reference	Mice	Sex	Intervention	Liver	Circulation	Intestine	Microbiome	Aorta
84	LDLR	NR	Quercetin		MDA, IL6	LPA, PEIPC, POVPC, PGPC, Cecal bile acid and CHO, coprostanol	F/B, Alpha diversity *Akkermansia, Bacteroides, Parabacteroides, Ruminococcus, Lactobacillus*	Plaque
77	ApoE	F	Resveratrol	FMO3	TMAO	Bile acid deconjugation FXR-FGF15	*Bacteroides, Lactobacillus, Bifidobacterium, Akkermansia* F/B, *Prevotella, Ruminococcaceae_unclassified, Biophila*.	Plaque
79	ApoE	F	Geraniin	FMO3	TMAO IL-10 IL-1β, IL-6, and TNF-α		*Bacteroides, Alloprevotella, Alistipes*	Plaque
86	ApoE	M	Curcumin (Cadmium)		TC, TG, LDL, HDL Cadmium TMAO		F/B *Lactobacillaceae*, Unspecified_S24_7, Lactobacillus, *Verrucomicrobia, Akkermansia*.	Plaque M2, M1 macrophages NF-kB, NLRP, IL1β, IL6
70	ApoE	M	Procyanidin A2	PPARγ, CYP7A1, ABCA1	TG, LDL, HDL MDA		F/B *Verrucomicrobia, Akkermansia unclassified_f__Prevotellaceae, Coriobacteriaceae_UCG-002*	Plaque Macrophages ICAM1, VCAM1
83	ApoE	F	Naringin	TBA, CHO TG, Cholic acid, TUDCA, UDCA, and glycocholic acid, PCSK9/IDOL CYP7A1/CYP7B1	TC, LDL, TBA, TMA, TMAO HDL, ox-LDL	FXR/FGF15	F/B Verrucomicrobia *Bacteroides, Bifidobacterium, Lactococcus*, and *Clostridium sensu_stricto_1 Eubacterium_fissicatena, Eubacterium_coprostanoligenes*, and *Eubacterium_brachy*	Plaque
56	ApoE	F	Gallic acid		TC, LDL, TG, IL3, IL12		F/B *Akkermansia and Dorea*	Plaque
56	ApoE	M	Gallic acid		TC, LDL, TG, IL3, IL12		F/B *Akkermansia Eubacterium fissicatena, Turicibacter and Dorea*	Plaque
68	ApoE	M	Berberine				F/B *Roseburia, Blautia, Allobaculum, Alistipes, and Turicibacter*	Plaque
69	ApoE	M	Berberine				F/B *Verrucomicrobia Proteobacteria*	Plaque collagen
80	ApoE	F	Berberine				*Lachnospiraceae* NK4A136, *Bacteroidales* S24-7 (unclassified), *Eubacterium*, Marvinbryantia, *Clostridiales unclassified, Ruminiclostridium 5,*	Plaque

							PrevotellaceaeNK3B31, Bifidobacterium	
81	ApoE	F	Berberine				*Verrucomicrobia Akkermansia Bacteroides*	Plaque
72	ApoE	M	*Gingko biloba*	FMO3	TC, TG, LDL, VLDL, hsCRP, HDL, glucose TMA, TMAO		F/B *Deferrobacters, Helicobacter, Roseburia, Bacteroides*	Plaque,
75	LDLR	M	*Gingko biloba*		TC, TG, LDL, MCP-1, IL-1β and TNF-α Primary BA (IAA) Secondary BA SCFAs	Claudin-1, ZO-1	F/B *Desulfovibrionaceae, Akkermansia, Alistipes, Rikenellaceae RC9 group, Alloprevotella and Parabacteroides Blautia, Lachnospiraceae, Lachnoclostridium, Colidextribacter, Faecalibaculum, Roseburia, Dubosiella, Erysipelatoclostridium and Ruminococcus torques*	Plaque CD63, CD36, SR-A1
82	ApoE	F	Tea (*Ligustrum robustum*)	FMO3, CHO SR-B1	TMA, TMAO BA	CHO, BA in feces SCFAs, NPC1L1, ABCG8	F/B *Lachnospiraceae_FCS020_group, Odoribacter* and *Oscillibacter*	Plaque
57	ApoE	M,F	Tea polyphenols		TC, LDL, HDL		*Bifodobacterium*	Plaque, collagen (plaque stability)
66	ApoE	M	Dingxin Recipe IV	LXR-α, SREBP1	TC, LDL, TG, MDA, LDH, SOD	Butyrate, propionate, acetate (feces)	F/B *Muribaculaceae, Ruminococcaceae, Erysipelotrichaceae, Ileibacterium and Allobaculum.*	Plaque
74	ApoE	M	Ophiopogo-nin D,	AST, ALT, steatosis, mTOR, SREBP1, SCD1	TC, LDL, TG, HDL, MDA, LDH, SOD Glucose tolerance, insulin resistance	leucine, acetate, ribose, propionate, valine, methionine, glutamate, lysine butyrate	F/B, diversity *Erysipelotrichaceae, Muribaculaceae, Faecalibaculum, Ileibacterium*	Plaque
85	ApoE	NR	Gypenoside XLIX	FMO3	TC, LDL, TG, HDL TMAO, MDA	Butyrate, propionate, acetate (feces)	F/B, alpha diversity, *Eubacterium, Roseburia, Bifidobacterium, Lactobacillus*, and *Prevotella Clostridioides and Desulfovibrionaceae*	Plaque
92	ApoE	M	TSG/PMRP		TC, TG, oxLDL, IL-6, TNF-α, VCAM-1, MCP-1, ICAM, CCR2		F/B, *Proteobacteria* and *Tenericutes, Helicobacter pylori, Akkermansia*	Plaque (quantification not provided)
93	ApoE	M	Qing-Xin-Jie-Yu Granule	CYP7A1, CYP27A1	TC, LDL, TG, HDL, IL-1β and IL-6	FGF15 and β-Klotho	*Roseburia, Aerococcus, Enterobacter, Defluviitaleaceae_UCG_011, Turicibacter, Papillibacter, Jeotgailcoccus, Ruminococcus, Alistipes, Rikenella, Blautia*	Plaque (necrotic core, SMC), macrophages, T-cells, IL-1β and IL-6
64	ApoE	M	Lingonberry	CYP7A1	TC, TG	Cecal propionate Total SCFAs	F/B, alpha-diversity	Plaque

							Akkermansia, *Parabacteroides* and *Clostridium, Blautia producta, Clostridum difficile, and Eubacterium dolichum*	
76	ApoE	F	Lingonberry (Whole LB, flavonoids and fiber extracts)		TC, TG (Whole LB and LB fiber) Creatinine, L-carnitine (whole LB), TMAO (flavonoids and fiber)		F/B, alpha-diversity *Akkermansia (Whole LB, flavonoid), Oscillospira, Lactobacillus, Mucispirillum and Bilophila while Bifidobacterium* *Flavonids: Lactobacillus and Bifidobacterium* *Fiber: unclassified S24_7 and Clostridiales*	Plaque
58	ApoE	M	Pomegranate juice		TC, TG		F/B *Lachnospiraceae* (*Coprococcus*), *Dehalobacteriaceae* family (*Dehalobacterium*) *Lactobacillaceae* family (*Lactobacillus*).	Plaque not assessed Lipid peroxidation In vitro in macrophages: SREBP2, HMGCR, and DGAT1
59	ApoE	M	Pomegranate and chitin glucan	TG, TNF-α, IL-1β and COX-2	TC, TG, (IL-6, IL-10, IL-1β, MIP1α, MCP1, TNF-α, sE-Selectin, sICAM-1, PAI-1, and proMMP-9,		*Akkermansia, Allstipes spp. and Lactobacillus spp*	Plaque not assessed endothelial dysfunction, eNOS and hb-NO in mesenteric arteries
61	LDLR	M	Oat fiber		Glucose, insulin resistance	Gut barrier function, Claudin-1, ZO-1 TLR4/ NFκB, NLRP3, caspace-1 Gut metabolites: L-tyrosine and niacinamide isobutyrylcarnitine, valerylcarnitine, 1-methylguanosine, and 2- methylguanosine	NR	Plaque TLR4/ NFκB, NLRP3, GPR19A
67	ApoE	M	Millet shell polyphenols		LPS, TNF-α, and IL-1β	Claudin-1, ZO-1	*Bacteroidetes, Oscillospira and Ruminococcus, Verrucomicrobia and Actinobacteria, Allobaculum*	Plaque TNF-α, and IL-1β
71	ApoE	M	Foxtail millet bran protein				*Firmicutes* *Lactobacillus and Turicibacter* *Ruminococcus, Allobaculum, Akkermansia*	Plaque
95	ApoE	M	Red yeast rice	HMG-CoA reductase	TC, LDL, TG, TNF-α, and IL-1β	Microvilli restored, of JAM-1 and occludin	*Firmicutes, Rikenellaceae, Alistipes, Barnesiella, and Flavonifractor* *Bacteroidaceae, Bacteroides and Anaeroplasma*	Plaque TLR2, TLR4, MAPK

65	LDLR	?	F&V mix	Steatosis TNF-α, Fasn	TG, VLDL, TC, LDL, TNF-α		*Diversity, Verrumicrobi Firmicutes, Bacteroides, and Actinobacteria, S24–7 and Clostridiales*	Plaque
73	ApoE	M	Astaxanthin (carotenoid)	TC, TG, Ampkα, Lxrα, Cyp7a1, Fxr, ABCG5/8, NPC1L1, ACTA2, MTTP	TC, TG, nonHDL CHO, glucose, HDL	,Ampkα, Lxrα, Cyp7a1, Fxr, JAMA, occluding,, Mucin-2 coprostanol and campesterol, CDCA, LCA, DCA, CA	F/B, *Verrucomicrobia,* ***Akkermansia****, Bacteroides, Oscillibacter, and Ruminiclostridun_9 Alloprevotella, Desulfovibrio, Muribaculum, Odoribacter, and Parabacteroides*	Plaque
62	ApoE	M	Brussels chicory		LPS, IL-1β and TNF-α.	Intestinal permeability, LPS	*Ruminococcaceae*	Plaque volume Plaque stability
63	ApoE	NR	Green coffee extract	Liver damage IL-6	insulin resistance, inflammation		*Mogibacteriaceae, Coprococcus, Dorea, Ruminococcus, Firmicutes, and Desulfovibrio*	Plaque
78	ApoE	F	Brown bean		Serum lipids, TMAO	Acetic acid, propionic acid, butyric acid	F/B Alpha diversity, *Actinobacteria* and *Bacteroidetes, of unclassified S24-7, Prevotella, Bifidobacterium, and unclassified Clostridiales, Lactobacillus*	Plaque (trended down)

Red denotes increases, blue decreases and black no change in expression.

BA: bile acid

LB: Lingonberry

F&V mix: fruit and vegetable mix

NR: Not reported
